# Novel Insights into the Biological Activity of *Croton lechleri* Twigs Extracts and Advancements in Their Sustainable Recovery

**DOI:** 10.3390/molecules29174161

**Published:** 2024-09-02

**Authors:** Alexander Tzintzarov, Stanislava S. Boyadzhieva, Jose A. P. Coelho, Flora Tsvetanova, Maria Petrova, Georgi Stoev, Dragomir S. Yankov, Iva Ugrinova, Roumiana P. Stateva

**Affiliations:** 1 Institute of Molecular Biology, Bulgarian Academy of Sciences, 1113 Sofia, Bulgaria; alexander_imb@abv.bg (A.T.); mhristova84@abv.bg (M.P.); georgi_stoev_imb@abv.bg (G.S.); 2 Institute of Chemical Engineering, Bulgarian Academy of Sciences, 1113 Sofia, Bulgaria; maleic@abv.bg (S.S.B.); florablue@abv.bg (F.T.); yanpe@bas.bg (D.S.Y.); 3 Instituto Superior de Engenharia de Lisboa, Instituto Politécnico de Lisboa, Rua Conselheiro Emídio Navarro 1, 1959-007 Lisboa, Portugal; jcoelho@deq.isel.ipl.pt; 4 Centro de Química Estrutural, Institute of Molecular Sciences, Instituto Superior Técnico, Universidade de Lisboa, Av. Rovisco Pais, 1049 001 Lisboa, Portugal

**Keywords:** *Croton lechleri* twig extracts, supercritical, pressurized liquid extractions, cytotoxicity, selectivity index, total phenolic and flavonoid content, antioxidant activity

## Abstract

Sangre de drago, the sap of *Croton lechleri* Müll. Arg. tree, has been used for centuries in traditional medicine owing to its diverse biological activities. Extracts derived from different parts of the species contain a multitude of phytochemicals with varied applications. Twigs, however, are among the least studied parts of the plant. This study unveils new biological activities of *Croton lechleri* twig extracts recovered by applying Soxhlet and advanced green techniques. For all extracts, total phenolic content and antioxidant activity were determined. Subsequently, four were selected, and their cytotoxic effects were assessed on both normal (HaCat) and malignant melanoma (A375) skin cell lines using the MTT assay and trypan blue exclusion assay. All showed dose-dependent cytotoxicity, with the Soxhlet ethanol extract demonstrating the highest selectivity towards A375 cells over HaCat cells. The extracts induced apoptosis and necrosis, as confirmed by Annexin V/PI dual-labeling and flow cytometry, highlighting their ability to trigger programmed cell death in cancer cells. The selective inhibition of cell cycle progression in A375 compared to HaCat observed both for Soxhlet ethanol and pressurized ethanol extracts induces cell cycle arrest at multiple points, primarily in the G1 and G2/M phases, and significantly reduces DNA synthesis as evidenced by the decrease in the S-phase population, confirmed by the EdU assay. Consequently, the Soxhlet extract composition was analyzed using LC-MS, which revealed their richness in polyphenolic compounds, particularly flavonoids from the flavonol subclass.

## 1. Introduction

*Croton lechleri* Mull. Arg., a species of flowering plant in the spurge family *Euphorbiaceae*, is a tree growing in Mexico, Colombia, Venezuela, Ecuador, Peru, and Brazil. It is best known for the production of characteristic thick deep red latex, called sangre de drago (dragon’s blood), which is secreted when the tree bark is cut and is considered one of the strongest healing agents known [1]. As reported by Gupta et al. [2], the Spanish Jesuit P. Bernabe Cobo was the first to report that the red resin was used widely throughout the native tribes of Mexico, Peru, and Ecuador in the 1600s. Early Greeks, Romans, and Arabs also used the latex for its medicinal properties.

For centuries, sangre de drago has been widely employed internally to treat intestinal complaints and externally as a popular cicatrizant for wound healing in holistic medicine [1,2]. In recent years, the results of extensive in vitro and in vivo studies have confirmed its anti-inflammatory, antibacterial, antiviral, antidiarrheal, anticancer, antirheumatic, antiseptic, and neuroprotective actions, activity against glycoxidation, and usefulness in ROS-related diseases of the tree sap [2]. Moreover, the data collected proved a correlation between the very rich chemical composition of the latex and bioactivities observed, which were directly associated with the presence of certain secondary metabolites such as diterpenes (clerodane, labdane, abietane, tigliane, entkaurane, and cembranolides), alkaloids, phenolic compounds, etc. [3].

For example, it was shown that over 90% of the dry weight of the dragon’s blood is composed of phenolic compounds—e.g., proanthocyanidins, flavonols, flavan-3-ols, etc.—which are potent antioxidants capable of considerably reducing the toxic effects of free radicals. Furthermore, flavonols can act as inhibitors of the enzymes involved in the biotransformation of precarcinogens. The latex also contains the alkaloid taspine, which exhibits cytotoxicity as a plant metabolite and is the active ingredient responsible for the anticancer and anti-inflammatory activities of the sap as well as for its wound-healing properties [4,5].

Even though *Croton* latex is the most widely investigated product, other parts of *Croton* plants were also studied. Data on the chemical composition and biological activity of extracts recovered applying different methods from the stem, bark, leaves, roots, and twigs of a considerable number of representatives of the *Croton* genus have been presented, summarized, and discussed in detail over the years in many publications and reviews, including some of the most recent ones, without the ambition to be extensive [6,7,8,9,10,11,12].

It should be noted that the essential oil of *Croton* plants is also of potential medicinal interest. For example, Rossi et al. [13] analyzed the distilled essential oil from the stem bark of *C. lechleri*, identifying significant concentrations of compounds contributing to the anti-bacterial, cytotoxic, and anti-mutagenic properties of the oil. Infusions of the bark, stem, and leaves of *Croton* species have been prepared and used in folk medicine for centuries to treat various ailments such as stomach pain, vomiting, diarrhea, hemorrhage, hemoptysis, and swelling [6]. Recently, conventional methods such as maceration, hydrodistillation, steam trapping, and organic solvent extraction have been employed to recover *Croton* plant extracts [7].

*Croton* genus is one of the most diverse in the *Euphorbiaceae* family, and the plant material of the species in general demonstrates substantial chemical complexity resulting from the type and quantity of bioactives present. Taking into consideration that the composition of extracts depends considerably on the techniques applied to recover them, further studies are needed to improve the extraction.

Hence, the focus has nowadays shifted to employing cutting-edge advanced methods with low environmental impact that guarantee the obtainment of high-quality extracts with controlled composition—techniques that are sensitive, selective, fast; use milder temperatures; and require considerably less extraction times. Moreover, since the application of organic solvents is circumvented, subsequent sophisticated separation methods to purify the compounds are not necessary. Such *Croton* extracts could possess considerable potential, with various novel perspectives for their application in the food, nutra-, and pharmaceutical industries.

Representatives of such innovative methods are microwave-assisted extraction (MAE), ultrasonic-assisted extraction, extraction with pressurized fluids such as supercritical fluid extraction (SFE), and pressurized liquid extraction (PLE). However, it should be underlined that their application to the recovery of *Croton* extracts till today is more than limited.

For example, Sousa et al. [14] advocated an optimized ultrasound-assisted extraction for the recovery of bioactive phenolic compounds from the leaves of *Croton heliotropiifolius* plants. Since the application of ultrasound increases the permeability of cell walls, the penetration of the solvent ethanol into the matrix is accelerated, resulting in an increased solubility of the bioactives.

Bezerra et al. [11] recovered extracts from *C. matourensis* Aubl. leaves applying supercritical CO_2_ (scCO_2_) and compared their yield, chemical composition, and antioxidant activity with those obtained using hydrodistillation and *n*-hexane. The authors also assessed their extracts with those reported in the study of Sousa et al. [15]. The latter used pressurized CO_2_, steam distillation, and ethanol to recover volatile oil from *Croton zehntneri* Pax et Hoff leaves. Liu et al. [16] studied the chemical composition and anti-proliferative, autophagic, apoptosis-inducing, and related molecular effects on A549 tumor cells of oils obtained using scCO_2_ from the roots of *Croton crassifolius* Geisel—a herb belonging to the Croton genus and cultivated in several Chinese provinces. Cevallos-Morillo et al. [17] applied supercritical antisolvent extraction to a lyophilized *C. lechleri* latex to obtain solid extracts for their characterization as novel green corrosion inhibitors of AB in hydrochloric acid media [17]. The authors used CO_2_ antisolvent extraction because the method alleviates some of the drawbacks of scCO_2_ extraction related to the limited capacity of CO_2_ to dissolve polar compounds.

PLE is yet another relatively new extraction method that is highly selective and efficient. It has been utilized to recover secondary metabolites and other value-added substances from a wide range of biological matrices; see for example [18,19]. However, the analyses of the available literature performed did not produce any results regarding the application of PLE to the recovery of extracts from a *Croton* species.

As an object of investigation in this work, *C. lechleri* twigs were chosen. It should be underlined that twigs are among the least-studied parts of the plant. In a recent review [7], only three contributions that discuss the isolation, identification, and bioactivity of novel compounds from twigs and leaves extracts recovered by methanol under reflux from *Croton* species growing in different Chinese provinces were referred to [20,21,22].

Still, as is known, pruning the *Croton* trees is important to promote the seedlings’ growth [23]. The biomass obtained is considered a waste that is underused and neglected to a certain extent. Yet, since twigs could be a sustainable storehouse of important phytochemicals and secondary metabolites, examination of the biological activity of their extracts will provide new information and be an invaluable addition to the existing gap.

In view of this, the main aim of our investigations is an analysis of cytotoxicity, apoptosis induction, and cell cycle perturbations, as well as the wound-healing potential of twig extracts obtained by conventional and two advanced methods—namely, Soxhlet and scCO_2_ extraction without and with co-solvent, and extraction with pressurized ethanol. Moreover, considering the sustainability of compressed fluids as solvents, their application could reveal previously unexplored options for recovery of extracts from *C. lechleri* twigs.

The choice as to which extracts among all recovered will form the “promising set” and will be subsequently analyzed for bioactivity is made applying three criteria—the qualitative indicator color, the quantitative indicative criteria TPC, and the antioxidant activity of the extracts. Consequently, the polyphenolic profile of the extracts that exhibit the highest bioactivity will be identified and characterized.

Based on the analysis of the articles devoted to *C. lechleri* published in the available literature, it can be ascertained that the present work is unique in revealing novel aspects of the bioactivity and chemical composition of twig biomass extracts obtained by applying different techniques.

Consequently, the results achieved will mark a viable path to *C. lechleri* twigs’ sustainable valorization employing holistic integrated approaches that target the development of new products for human welfare and beyond.

## 2. Results and Discussion

### 2.1. Extraction Yield

#### 2.1.1. Soxhlet Extraction

Information about the extraction conditions and the yields achieved is given in Table 1.

In all cases, the extraction yield was calculated according to

(1) $$Yield\;\left(\%\right)=\frac{mass\;of\;extract\;\left(g\right)}{mass\;of\;sample\;\left(g\right)}\times 100\;$$

The lowest yield obtained is the one with the nonpolar n-hexane, which is expected. However, the solvent with the highest polarity water did not achieve a high yield. It is not only over 2 times lower than Soxhlet ethanol but also about 1.5 times lower than the second step Soxhlet ethanol. One possible explanation for this result is that alcohols are better solvents for alkaloids than water. Hence, both one- and two-step Soxhlet ethanol extractions render higher yields.

#### 2.1.2. Extraction with Neat scCO_2_ and with a Co-Solvent

As highlighted above, the number of investigations devoted to the application of pressurized fluids for the recovery of extracts from different parts of *Croton*, and *C. lechleri* in particular, are very limited. Bezerra et al. [10] applied two temperatures—40 and 50 °C, and four pressures—100, 200, 300, and 400 bar, to obtain extracts from *C. matourensis* leaves with neat scCO_2_. The yield varied in the range 1.49–5.60%, the lowest being registered at 50 °C and 100 bar and the highest at the same temperature but at the highest pressure applied. In a much earlier work [15], where also neat pressurized CO_2_ was used to obtain extracts from *C. zehntneri* leaves, the authors reported that the highest yield, 3.8%, was achieved at 20 °C and 66.7 bar. In [16], the scCO_2_ extraction yield of *Croton crassifolius* Geisel root at 25 MPa and 35 °C was reported to be 3.41%. The antisolvent supercritical extraction of lyophilized *C. lechleri* sap gave 1.84 wt % of a pink solid.

**Figure 1:**
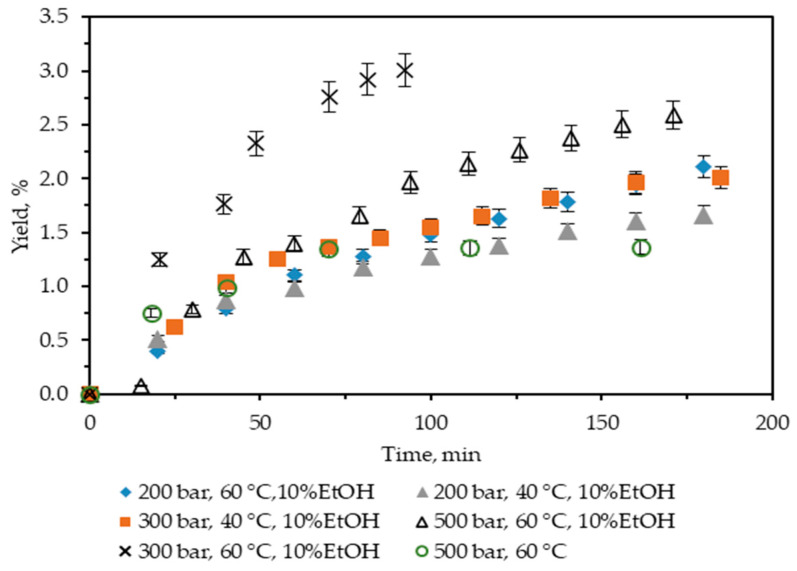
Cumulative extraction yield curves representing the influence of temperature, pressure, and co-solvent on *C. lechleri* yield as a function of time.

In the present study, first, neat scCO_2_ at *T* = 60 °C and *p* = 500 bar was used, and the yield was 1.36%. It is almost twice as low as Soxhlet *n*-hexane, so further extractions with scCO_2_ were not performed. Next, the influence of the addition to scCO_2_ of 10% ethanol as a co-solvent was studied, and the results obtained are summarized in Figure 1. It shows the cumulative extraction yield curves representing how the operating parameters temperature and pressure affect *C. lechleri* twig yield. Two temperatures (40 and 60 °C) and three pressures (200, 300, and 500 bar) were used, and the yields were in the range 1.67–3.0%.

All extracts obtained were green.

Regarding temperature, at a constant pressure, higher temperatures increase the yield. Thus, at 200 bar, a rise in the yield value from 1.66 to 2.11% is observed for 40 and 60 °C, respectively. In a complete analogy, the yield at 300 bars increases from 2 to 3%. However, for 200 bars, the trend in the increase of the cumulative yield, as represented by the extractions curves, is smooth in time, with the maximum reached approximately after 170 min of extraction. At 300 bar and 60 °C, the process is quite fast, the extraction curve is steep, and the maximum yield is achieved in less than 100 min.

If the influence of pressure on the yield is examined, the trend is positive at 40 °C—the yield increases from 1.66 to 2%, with the pressure increasing from 200 to 300 bars.

However, at 60 °C, the trend is more complex, goes through a maximum, and is far from straightforward to be easily generalized—the yield initially increases from 2.11 to 3, then falls to 2.59% for 200, 300, and 500 bars, respectively. Furthermore, the 300 bars and 60 °C case not only stands out as the one for which the highest yield is registered, but it is also achieved for the shortest extraction time. It could be hypothesized that the observed trend is a consequence of the presence of certain compounds in the biomass, for which a combination of a higher temperature (60 °C) and pressures not much higher than 300 bars is more favorable. Thus, not only is the yield increased, but the extraction time is also considerably shortened.

#### 2.1.3. Extraction with Pressurized Ethanol

Though PLE has proved itself to be a reliable green and efficient technique for the recovery, particularly of bioactives and secondary metabolites with thermo-sensitive structures, to the best of the authors’ knowledge, it has not been applied until present to obtain extracts from any part of a *Croton* species.

In this study, extraction with pressurized ethanol was performed at two temperatures, 40 and 60 °C. The pressure was maintained at 200 bar, which ensured that the solvent was in a liquid state. Extraction time was fixed to 200 min in order to provide a basis for comparison with the results obtained using scCO_2_ + 10% ethanol as a co-solvent. The two extracts recovered were red.

**Figure 2:**
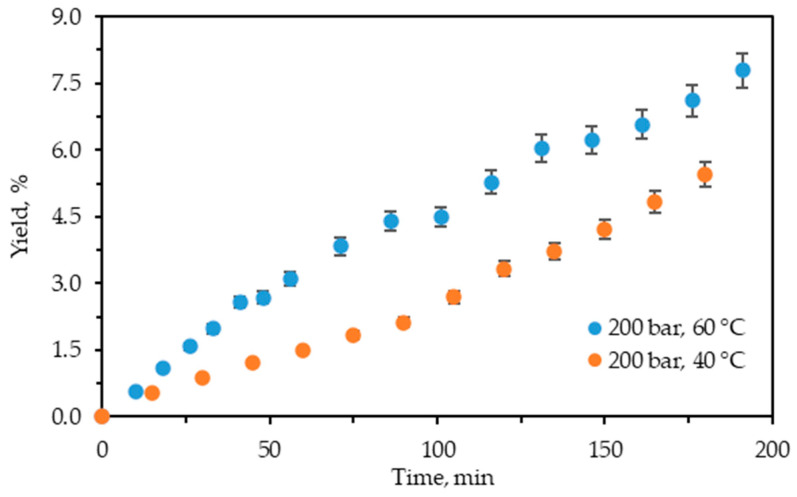
Cumulative extraction yield curves representing the influence of temperature at 200 bars on *C. lechleri* yield as a function of time.

Figure 2 displays the cumulative experimental kinetic extraction curves plotted vs. the extraction time for *C. lechleri* twigs.

The kinetic curves demonstrate that the process is still in the first phase, where the extraction rate is constant and at its highest value. The restriction of time to 200 min did not allow it to reach the diffusion-controlled stage and be completed.

As shown, the influence of temperature on the extraction yield is positive, analogously to the case of scCO_2_ + 10% ethanol extraction at 200 bars. The increase in the cumulative yield value with the increase in temperature for both techniques is also similar. However, the expanded ethanol extraction yields are higher than those for the scCO_2_ + 10% ethanol. Thus, at 40 °C the yield of the former is approximately 3.3 times higher (5.46% vs. 1.66), while at 60 °C it is about 3.7 times higher (7.79% vs. 2.11%) than those of the latter. Hence, pressurized ethanol extraction is more efficient with regard to yield than scCO_2_ + 10% ethanol.

With regard to extract color, the extracts obtained using Soxhlet water, Soxhlet ethanol (Table 1), and pressurized ethanol are all red, as is the sap of *C. lechleri*. Those recovered using neat scCO_2_ and scCO_2_ + ethanol are all green, regardless of the temperature and pressure applied. Hence, at this stage, it could be assumed that the red extracts might have higher total phenolic content and better antioxidant activity (expressed via IC50) than the green ones. The hypothesis is tested in the next section.

### 2.2. Total Phenolic Content (TPC) and Antioxidant Activity (AA)

The TPC and AA of the extracts obtained are shown in Table 2.

As is well known, TPC indicates the total phenol quantity in a given extract but does not provide any information about its specific composition. Still, the hypothesis advocated in the previous section finds some confirmation in the results obtained. Thus, all extracts with a red color have higher TPC than those with green. For example, the TPC values of pressurized ethanol extracts are about 7.4 to 9.5 times higher than those of scCO_2_ + ethanol at the same temperature and pressure.

The highest TPC value—248.78, was registered in the extract recovered by pressurized ethanol at 200 bar and 40 °C. Traditionally, it is considered—see for example [24]—that TPC is highest at an extraction temperature of 60–80 °C. Our results show that at 60 °C, the TPC of the pressurized ethanol extract is lower than that at 40 °C (199.92 vs. 248.78), which is analogous to the results for scCO_2_ + ethanol at 300 bar, but in contrast to those at 200 bar. Though the latter are practically commensurable, the one at 60 °C is higher. Of course, the Folin–Ciocâlteu method for calculating the TPC does not give any insight into the type of phenols present in the extracts recovered but accounts for all phenols and their degraded products. Furthermore, several studies have demonstrated that a high phenolic yield was achieved by applying PLE at temperatures well above 100 °C [24]. Moreover, Ibanez et al. showed that in PLE, polar phenolic compounds were extracted at low temperatures, and the less polar ones at higher temperatures [25]. Consequently, it is far from easy and trivial to propose a well-substantiated explanation of how extraction temperature influences polyphenol recovery.

The second- and third-highest TPC values were registered for the red extracts obtained by one- and two-step Soxhlet ethanol. The latter is practically commensurable with the PLE with EtOH at 200 bar and 60 °C.

However, one very important exception among the red extracts stands out and should be noted. It refers to the TPC calculated for the Soxhlet water extract, with a value not only the lowest among those of the red extracts, but actually almost two times lower than that of the expanded ethanol at 40 °C. It could be assumed that though water recovers some flavonoids (e.g., proanthocianidins that contribute to the red color), their quantity is still not sufficient to substantially increase the TPC. The reason could be that most flavonoids have low to medium polarity, for which ethanol is a better solvent.

The TPC values for all green extracts were very low, with the worst being determined for the Soxhlet *n*-hexane, which was to be expected.

Table 2 also shows the values of the IC50 parameter, which is used to determine the AA. IC50 represents the concentration of an antioxidant that inhibits 50% of free radicals by DPPH and, in our case, is expressed as mg dry extract per liter. The AA is categorized as very strong, moderate, and weak when the IC50 values are lower than 50, in the range 50–100, and higher than 150 µg·mL^−1^, respectively. The extract obtained using pressurized ethanol at 40 °C exhibits the best (lowest) IC50 value: 135.17. Though this is an expected result, considering that the highest TPC is calculated for it, the extract AA is still moderate. However, a somewhat unforeseen but interesting result is that the second-best performer is the Soxhlet water with IC50 = 145.83.

For both one- and two-step Soxhlet ethanol, the IC50 values are higher, and their AA can be categorized as moderate to low. As briefly commented above, though water is not the best solvent for flavonoids (compared to alcohols), it still recovers other bioactives with antioxidant activity. The weakest AA exhibits the extract obtained using pressurized ethanol at 60 °C, whose IC50 is the highest among all red extracts.

All green extracts exhibit very weak AA (very high IC50 values), which was anticipated taking into consideration their very low TPC. Though it is difficult to directly compare the results of other authors to those obtained by us, we should still mention the work of Bezerra et al. [10], where data on the TPC and IC50 of *C. matourensis Aubl*. leaf extracts recovered using neat scCO_2_ were reported. It was indicated that extracts with higher antioxidant activity (lower IC50 values) were those obtained at lower temperature and pressure—40 and 50 °C, and 100 bar. In our case, for the neat scCO_2_ and scCO_2_ + 10% ethanol green extracts, the lowest IC50 value was calculated for the extract obtained at 60 °C and 300 bar.

It is generally difficult to evaluate the combined impact of a wide variety of diverse parameters on the TPC and AA and establish a viable and sound explanation for the interrelation between TPC and antioxidant activity (IC50). For example, plant phenolic content varies depending on its genotype and growing conditions. Consequently, different types of phenols are recovered from the respective biomass. Also, the way specific polyphenols are bound to the plant tissue also varies, and as a result, the optimum extraction temperature is different from case to case; polyphenol thermal degradation occurs at dissimilar temperatures, etc. [24].

The TPC and IC50 values calculated for all extracts obtained using the three techniques applied in our study were used as a basis for the selection of a set of “extracts with potential”. It comprises four of the red extracts—namely, Soxhlet water, one-step ethanol, and both PLE ethanol. The 200 bar, 60 °C expanded ethanol was preferred to the second-step Soxhlet ethanol, though its TPC and IC50 were slightly worse than those of the latter.

In what follows, the total flavonoid content (TFC) of the four extracts will be determined first, followed by analysis of their bioactivity.

### 2.3. Total Flavonoid Content

The TFC of the four extracts chosen is displayed in Table 3.

Like TPC, TFC does indicate the presence of a whole class of natural compounds, but does not provide information about the particular content of an extract. Our results show that flavonoids in *C. lechleri* twig extracts are recovered in considerable concentrations when the solvent used is ethanol.

Most flavonoids in plants are found in their glycosylated form [26], in which they are more soluble in solvents with a lower polarity than water, such as ethanol [27]. Hence, it is understandable why the lowest TFC is determined in the Soxhlet aqueous extract, with a value at least three times lower than those of the three ethanol extracts.

Furthermore, the TFC trend observed differs slightly from that displayed by the TPC (Table 2). Thus, the highest TFC values are registered for pressurized ethanol at 40 °C, followed by that at 60 °C. Obviously, PLE performs better than Soxhlet ethanol with regard to the recovery of flavonoids, particularly those that are thermolabile. Another interesting observation is that higher extraction pressures applied enhance TFC (PLE with EtOH vs. Soxhlet), as reported by other authors as well [28,29]. Still, in our case, the influence of pressure is not that pronounced—the highest TFC of PLE with EtOH extract at 40 °C, 200 bar, is just 1.4 higher than that of Soxhlet ethanol.

### 2.4. Bioactivity Results

#### 2.4.1. Cytotoxicity

The cytotoxic effects of the four red extracts Soxhlet H_2_O, Soxhlet EtOH, and PLE with EtOH at 40 °C and 60 °C were investigated on the human normal keratinocyte cell line HaCat and the human malignant melanoma cell line A375, both originating from the skin. These two cell lines were selected because *Croton* latex is widely used in folk medicine for treating wounds and fractures, without any side effects in clinical studies [6], and there is also evidence that *C. lechleri* and taspine could inhibit cell proliferation with higher potency against melanoma SK23 cells, supporting the empirical use of the sap as an anticancer drug in ethnomedicine and taspine as a possible anticancer agent [5]. These data led us to focus on cell lines of skin origin.

**Figure 3:**
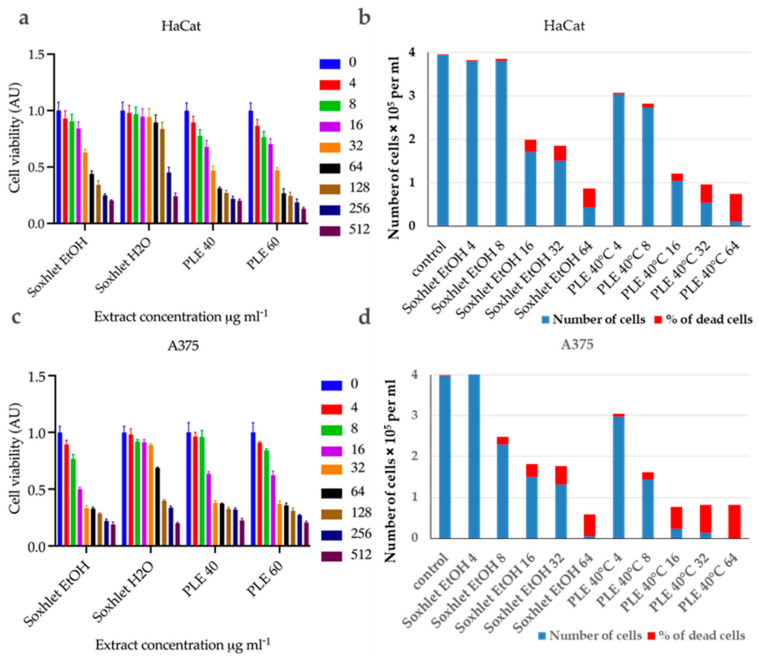
Summary bar graphs illustrating cell viability fraction (AU) for (**a**) HaCat and (**c**) A375 cells incubated with Soxhlet EtOH, Soxhlet H_2_O, and PLE with EtOH 40 °C and 60 °C extracts at concentrations ranging from 0 to 512 μg·mL^−1^, as indicated in the graph legend. Data are presented as the mean ± SD of three independent experiments carried out in quadruplicate. Bar graphs illustrate the results from the trypan blue exclusion assay carried out after a 24 h incubation of cells with Soxhlet EtOH and PLE with EtOH 40 °C extracts at concentrations ranging from 0 to 64 μg·mL^−1^ for (**b**) HaCat and (**d**) A375 cells. The data are presented as the mean of three independent experiments.

First, the cell viability was tested using the standard MTT assay. The MTT assay measures cellular metabolic activity as an indicator of cell viability. This analysis led to the establishment of dose–response relationships and the calculation of half-inhibitory concentrations (IC_50_) (the dose–response curves are presented in Supplementary Figure S1) (refer to Table 4). The experimental data (Figure 3) were normalized as fractions of the untreated control and analyzed using nonlinear regression analysis with GraphPad Prism 10 software. All tested extracts exhibited dose-dependent cytotoxicity, showing variability in intensity based on the specific cell line. Interestingly, the normal keratinocytes (Figure 3a) were found to be less sensitive compared to the malignant melanoma cell line A375 (Figure 3b).

HaCat Cells: The Soxhlet EtOH extract showed an IC50 (half maximal inhibitory concentration) of 63.47 μg·mL^−1^, indicating moderate cytotoxicity. The PLE with EtOH extracts had lower IC50 values, with 33.98 for the extract recovered at 40 °C and 30.50 μg·mL^−1^ for the one at 60 °C, respectively.

A375 Cells: The Soxhlet EtOH extract exhibited a significantly lower IC50 of 13.31 μg·mL^−1^, indicating high cytotoxicity. The PLE with EtOH extracts also showed potent cytotoxicity, with IC50 values of 18.91 μg·mL^−1^ for the extract obtained at 40 °C and 17.38 μg·mL^−1^ for the one at 60 °C. The selectivity index (SI) is the ratio of IC_50_ values for HaCat cells to A375 cells (refer to Table 4). A higher SI indicates greater selectivity towards cancer cells.

The Soxhlet EtOH extract had the highest SI, of 4.7, followed by 1.8 and 1.75 for PLE with EtOH at 40 °C and 60 °C, respectively. This suggests that the Soxhlet EtOH extract is more selective for cancer cells.

The viable and nonviable cell populations were also determined using the trypan blue exclusion assay. This assay distinguishes between viable and nonviable cells based on membrane integrity. The results mirrored those of the MTT assay, showing a concentration-dependent increase in nonviable cells with both extracts. At 24 h after treatment with different concentrations of Soxhlet EtOH and PLE with EtOH 40 °C extracts, the results indicate that the number of viable cells decrease for both the tested cell lines in a concentration-dependent manner, while the number of nonviable cells increase (refer to Figure 3c,d). Taken together, the data suggest that Soxhlet EtOH and PLE extracts exhibit antiproliferative and cytotoxic activity against both HaCat and A375 cells. This effect is more pronounced in A375 cells, which once again supports the observed selectivity of the extracts. Furthermore, the data from the trypan blue exclusion assay are consistent with the results of the MTT analysis. The highest selectivity index (SI) was observed at lower doses of 4 and 8 μg·mL^−1^, with an increase in concentration leading to higher toxicity and reduced selectivity. These results are highly promising, as they indicate the pronounced selectivity of the tested extracts, which is one of the most sought-after and challenging effects of anti-tumor therapy. In addition, a morphological analysis of cell shape alterations was conducted using an inverted phase-contrast microscope (200X). It was observed that the untreated cells displayed typical shapes with distinct outlines. In contrast, cells treated with Soxhlet EtOH or PLE with EtOH 40 °C extracts showed inhibited growth, a rounded morphology, loosened intercellular connections, reduced and slowed proliferation, and an increased number of floating dead cells (refer to Supplementary Figure S2). Similar results have been reported after a broad cytotoxicity screening of methanolic extract from *C. lechleri* leaves performed on a panel of cancer cell lines and HaCat cells as a normal control. HPLC analysis of the extract revealed two flavonoids—rutin and vitexin—as the major components. The results of the MTT assay showed cytotoxic properties of the extract towards HeLa and SKOV-3 cells and very poor toxicity against HaCat cells. The study also showed an apoptosis-inducing effect of the extract [30].

#### 2.4.2. Wound Healing Potential

As mentioned earlier, sangre de drago has been widely used as a popular remedy for promoting wound healing in holistic medicine. The process of wound healing is a complex series of events that can be broadly categorized into four phases: (i) coagulation and hemostasis; (ii) inflammation; (iii) proliferation, which involves key healing activities; and (iv) wound remodeling, which includes the formation of scar tissue [31,32]. To assess the potential impact of red twig extracts from the plant on wound healing, we conducted in vitro wound healing assays. One commonly used in vitro model for studying wound healing involves inducing mechanical damage to confluent cell layers, known as the “scratch assay” [33,34]. This method allows for the direct observation and measurement of cell migration and regeneration of the cell layer [32,34].

**Figure 4:**
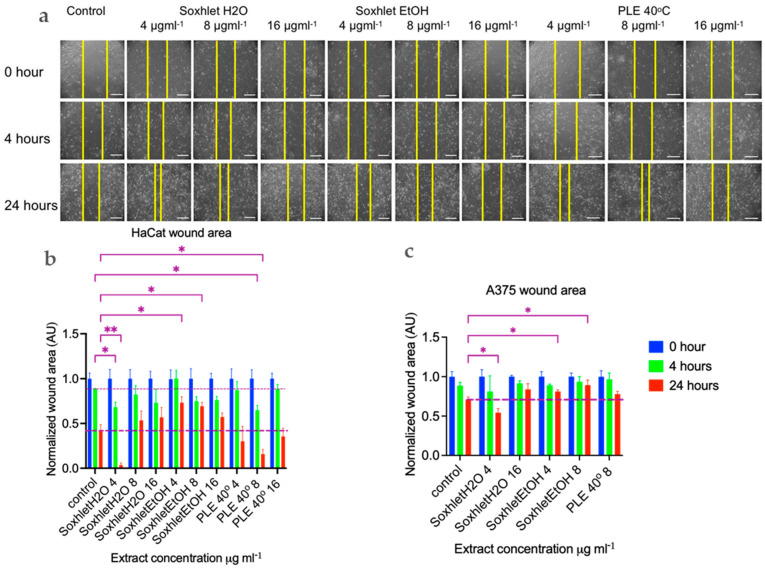
Cell migration (scratch wound healing assay). HaCat cells were seeded in 12-well plates at a density of 5 × 10^4^ cells per well, and a 20 µL pipette tip was used to create uniform wounds. Images were recorded at 4 and 24 h after wounding. (**a**) Representative images are shown from two independent experiments carried out in duplicate. The scale bar is 400 µm. Summary bar graph illustrating fraction wound closure at indicated time points during the scratch wound assay for (**b**) HaCat and (**c**) A375 cells incubated with Soxhlet EtOH, Soxhlet H_2_O, and PLE with EtOH 40 °C extracts with concentration in μg·mL^−1^ as indicated. The areas lacking cells were calculated using ImageJ (wound healing size tool plugin, ImageJ). Data are presented as the mean ± SEM of two independent experiments carried out in duplicate. Statistical analysis was performed using two-way ANOVA with post hoc analysis using Tukey’s multiple comparison test. Probability values were considered significant at * *p* < 0.05 and ** *p* < 0.01 vs. control.

The first migrating cells were observed after four hours (see Figure 4). The progress of wound healing is depicted in Figure 4a,b, which shows the normalized scratch wound area in arbitrary units [AU]. The Soxhlet water [4 μgmL^−1^] and PLE 40 °C [8 μgmL^−1^] extracts exhibited a stimulating effect on wound closure, evident at 4 h with the wound closing 20% and 24% more compared to the control, with corresponding *p* values of 0.04 and 0.02, respectively. Statistical analysis was conducted using a two-way ANOVA, followed by Tukey’s multiple comparisons test (refer to Figure 4). By 24 h, the trend towards more complete wound closure with these two extracts persisted. The effect of the Soxhlet water extract intensified to 38%, while that of the PLE 40 °C extract remained nearly constant at 25% in comparison with the control wound area at the same time point. Notably, when treated with the Soxhlet EtOH [4 μg·mL^−1^] and Soxhlet EtOH [8 μg·mL^−1^] extracts, two additional statistically significant findings emerged. However, in this case, the effect was reversed, leading to a suppression of the process by 32% and 28%, respectively. This finding was intriguing from another perspective, as it pertains to inhibiting the motility and/or proliferation of cancer cells. Wound healing is a complex cellular and biochemical process essential for repairing structurally damaged tissue, but also in cancer invasion and metastasis. In the cytotoxicity tests, apart from normal human keratinocytes, we also employed A375 malignant melanoma cells. The results revealed that these cells exhibited greater sensitivity to treatment with all extracts, including Soxhlet H_2_O, compared to normal keratinocytes HaCat. It was intriguing to investigate whether the Soxhlet EtOH [4 μg·mL^−1^] and Soxhlet EtOH [8 μg·mL^−1^] extracts could also inhibit the cell motility of A375 cells. As shown in Figure 4c, no effects were observed at 4 h, but at 24 h, it was noted that the Soxhlet H_2_O extract intensified to 16%, while the effect of the PLE 40 °C extract remained statistically insignificant. Treatment with the Soxhlet EtOH [4 μg·mL^−1^] and Soxhlet EtOH [8 μg·mL^−1^] extracts resulted in a statistically significant and concentration-dependent suppression of the process by 10% and 18%, respectively.

#### 2.4.3. Annexin V Staining Assay

**Figure 5:**
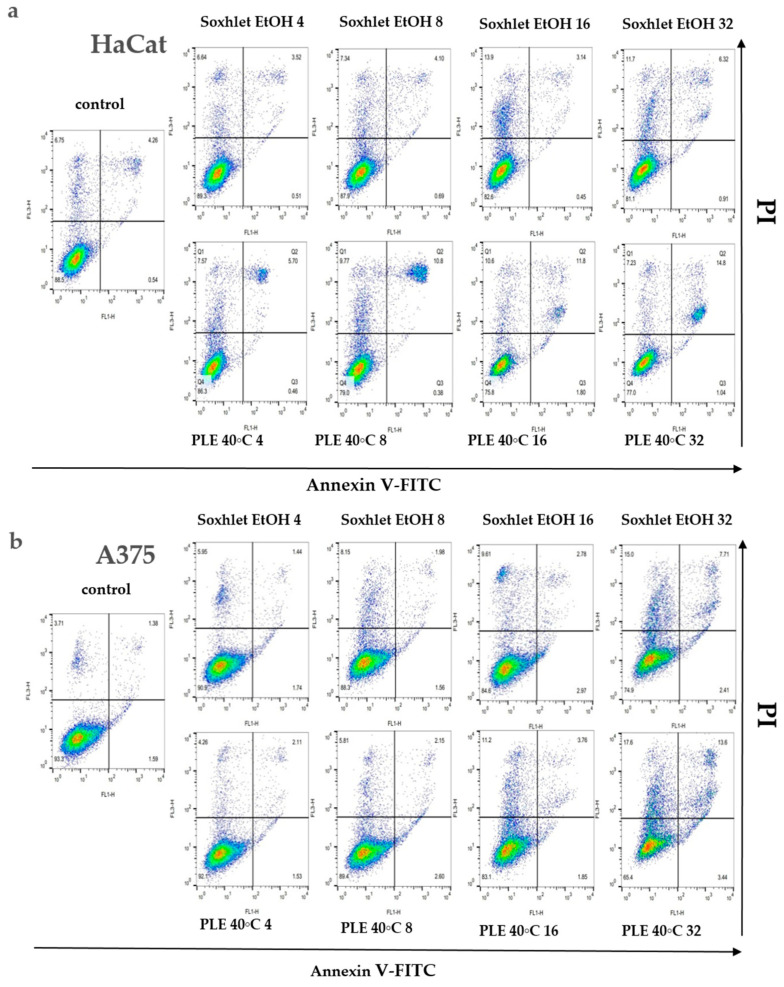

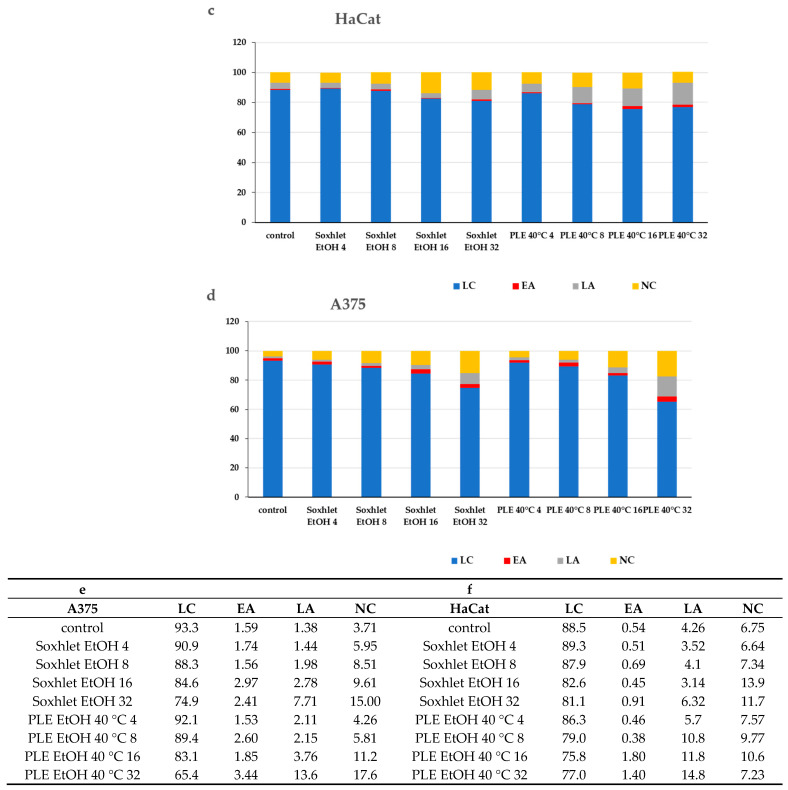
Results from the Annexin V-FITC/PI staining for apoptotic cells after 24 h treatment with Soxhlet EtOH and pressurized ethanol at 40 °C with concentrations ranging from 4 to 32 µg·mL^−1^; (**a**,**b**) Graphs representing the distribution of cells using Annexin V-FITC/PI fluorescence intensity; (**c**,**d**) Bar plots representing the percentage of living (LC), early apoptotic (EA), late apoptotic (LA), and necrotic (NC) cells of the total cell count; (**e**,**f**) Numeric values of the plot are shown in the tables. Data are presented as the mean ± SD of three independent experiments.

An investigation into the cellular demise triggered by Soxhlet EtOH and pressurized ethanol extracts was undertaken through Annexin V/propidium iodide (PI) dual-labeling. Annexin V binds to phosphatidylserine, an epitope exposed on the external membrane during the early stages of apoptosis, while PI highlights the DNA of deceased cells. The analyzed extracts, comprising diverse constituents, manifest intricate and multifaceted effects. Flow cytometry analysis elucidated that Soxhlet EtOH and PLE with ethanol extracts elicit apoptotic and necrotic phenomena in both tested skin cell lines, with intensification correlating with escalating concentrations. Investigating the levels of apoptosis and necrosis in cells cultivated in the presence of Soxhlet EtOH and pressurized ethanol extracts provides initial insights into the mechanism of cell death induced by the extracts in the two cell lines tested. For this analysis, the cells were treated with concentrations of the extracts at 4, 8, 16, and 32 μgmL^−1^ for each cell line. The samples were collected and analyzed at the 24th hour. Discrepancies in allocating specific fractions across varying conditions imply distinctive modes of action for each extract. A 24 h exposure to both extracts showed a notable surge in the proportion of early and late apoptotic HaCat (see Figure 5a) and A375 cells (see Figure 5b). Once more, the concentration-dependent response and selective effect were conspicuous, with the A375 cell line displaying heightened sensitivity to the treatment. Intriguingly, the pressurized ethanol extract induced nearly twofold more apoptosis in both cell lines (early apoptosis + late apoptosis) despite a mere 20% rise in overall cell death (early apoptosis + late apoptosis + necrosis). The extracts likely induce cytotoxicity through multiple mechanisms, including the induction of apoptosis and the disruption of cell membrane integrity. The increase in early and late apoptotic cells suggests the activation of apoptotic pathways, potentially through mitochondrial dysfunction or activation of death receptors. The higher selectivity index for A375 cells suggests that the extracts preferentially target cancer cells over normal cells. This selectivity is crucial for minimizing side effects in potential therapeutic applications. The differential cytotoxicity observed in normal versus cancer cells may be due to differences in metabolic activity, cell membrane composition, or the presence of specific receptors targeted by the bioactive compounds in the extracts.

The subsequent chemical analysis of the extracts revealed that most of the constituents are of a polyphenolic nature—carbohydrate derivatives of gallic acid, and modifications of the flavonol quercetin (see Section 2.5). Previous studies of compounds with similar structures suggest that galloyl modifications of sugars and flavonoids induce properties, similar to the ones observed in our results. A DNA fragmentation assay performed on U937 cells revealed that the presence of a galloyl group in the structure of the B-ring of flavanols enhances the apoptosis-inducing activity of these compounds [35].

#### 2.4.4. Cell Cycle Analysis

**Figure 6:**
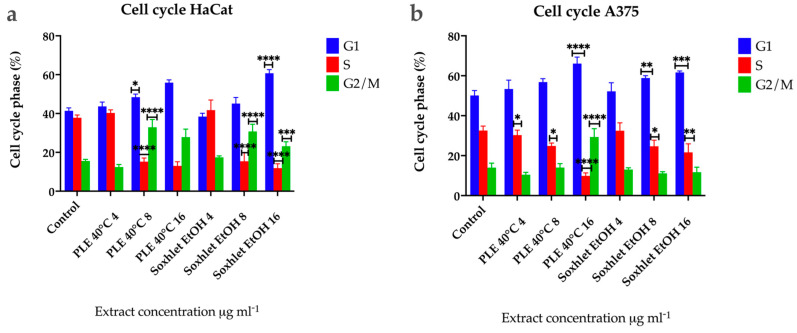
Results from the cell cycle progression analysis using flow cytometry of (**a**) HaCat and (**b**) A375 cells after 24 h treatment with Soxhlet EtOH and PLE 40 °C extracts with concentrations ranging from 4 to 16 µg·mL^−1^. Data are presented as the mean ± SD of three independent experiments carried out in duplicate. Statistical analysis was performed using two-way ANOVA with post hoc analysis using Tukey’s multiple comparison test. Probability values were considered significant at * *p* < 0.05, ** *p* < 0.01, *** *p* < 0.001, and **** *p* < 0.0001.

To further define the inhibitory effect of Soxhlet (EtOH) and pressurized ethanol extracts, we investigated their effect on the cell cycle by flow cytometry at 24 h using an FL2 channel at 575 nm and a speed of up to 600 cells per second. The cells analyzed were not synchronized beforehand, resulting in varying cell numbers in each phase. The distinct profiles observed in the two cell lines indicate differences in the progression rates of the cell cycle (Figure 6). The results revealed that Soxhlet EtOH and pressurized ethanol extracts impact the cell cycle of both cell lines similarly. During the time course treatment with the extracts, cell cycle analysis demonstrated a progressive and significant rise in the cell population with 2n DNA content for both cell lines. Specifically, the percentage of cells in G1 increased in untreated populations from 42% for HaCat (Figure 6a) and 50% for A375 (Figure 6b) to 56.9% and 61% in populations treated with 16 µg·mL^−1^ PLE with EtOH extract, and to 66% and 62% with 16 μg·mL^−1^ Soxhlet EtOH extract over 24 h, respectively. The accumulation of cells in the G1 phase indicates the activation of the G1/S checkpoint. This checkpoint serves as a critical control mechanism to prevent the replication of damaged DNA. The extracts appear to activate this checkpoint, halting cell cycle progression and preventing cells from entering the S phase. A similar trend was observed in the population with 4n DNA content (G2/M phase), with an increase from 12% for HaCat and 14% for A375 in the untreated population to 28% and 23% in the populations treated with 16 μg·mL^−1^ PLE with EtOH extract. In the case of Soxhlet EtOH extract treatment, HaCat cells showed a rise to 29%, while A375 did not exhibit a statistically significant difference compared to the untreated control (12%) when treated with 16 µg·mL^−1^ Soxhlet EtOH extract for 24 h. The increase in the G2/M phase population suggests that cells entering G2 are unable to proceed to mitosis, potentially due to the activation of the G2/M checkpoint. This could be a response to DNA damage or incomplete DNA replication, which the cells detect, leading to cell cycle arrest. The cell cycle analysis also revealed a significant decrease in the number of cells in the S phase for both cell lines, from 37.5% to 13.4% with 16 µg·mL^−1^ PLE and 11.7% with 16 µg·mL^−1^ Soxhlet EtOH for HaCat, and from 33% to 9.8% and 21% for A375, respectively. This alteration in the S-phase population upon treatment with Soxhlet EtOH and pressurized ethanol extracts was further confirmed by the 5-ethynyl-2′-deoxyuridine (EdU) assay. The most notable disturbance in the cell cycle is characterized by a reduction in the number of cells in the S phase and an increase in the other two phases. This observed effect may be related to the activation of the G1/S checkpoint due to the treatment, leading to the accumulation of cells in the G1 phase. One possible explanation for the increased peak in G2/M phase in HaCat cells is that cells that entered G2 before treatment fail to complete mitosis or cytokinesis or the G2/M checkpoint is activated. Furthermore, increasing treatment dosage correlated with an increased population in the sub-G1 phase, indicating the induction of apoptosis for both cell lines. This was supported by the Annexin V/PI staining results, which show an increase in apoptotic cells.

As mentioned in the introduction of this paper, *Croton lechleri* is a tree with a long history of medicinal use. Previous phytochemical analyses of this plant’s content have identified proanthocyanidins, alkaloids, phenols, flavonols, diterpenes, and essential oils [36]. *Croton lechleri* is most well known for the application of its sap, sangre de drago, which apart from other illnesses has been used for traditional cancer treatment as well. This effect of the sap has been tested in several in vitro studies, but single active compounds have not been identified [37,38]. Other studies have suggested taspine, an alkaloid found in the sap, as the key component in the context of cytotoxicity. Taspine isolated from the sap of *C. palanostigma* has been found to be toxic against V-79 lung cells and KB papilloma cells. [39]. A deeper analysis of the effect of the *Croton lechleri* sap on SK23 melanoma cells and HT29 colorectal adenocarcinoma cells, comparing its toxic effect with taspine alone and two chemotherapeutic drugs—paclitaxel and vinblastine—has been conducted. The analysis revealed that treatment with the sap induced a cell cycle arrest in the G0/G1 phase of the cell cycle, and with an increase in the concentration of the treatment, the arrest of the cell cycle happened in sub-G1, along with a strong decrease in the S phase for all the treatment concentrations. No such effect has been observed in the treatment with taspine [5]. This observation leads to the hypothesis that other constituents of the tree sap have cytotoxic and particular antitumor effects.

#### 2.4.5. 5-Ethynyl-2′-Deoxyuridine (EdU) Labeling

The 5-ethynyl-2′-deoxyuridine (EdU) assay is a widely used technique to measure cell proliferation. EdU is a thymidine analog that is incorporated into newly synthesized DNA during the S phase of the cell cycle. The incorporated EdU can then be detected using a fluorescent azide via a click chemistry reaction, allowing for the visualization and quantification of cell proliferation. This assay is particularly useful in assessing the effects of various treatments on cell cycle progression and DNA synthesis.

**Figure 7:**
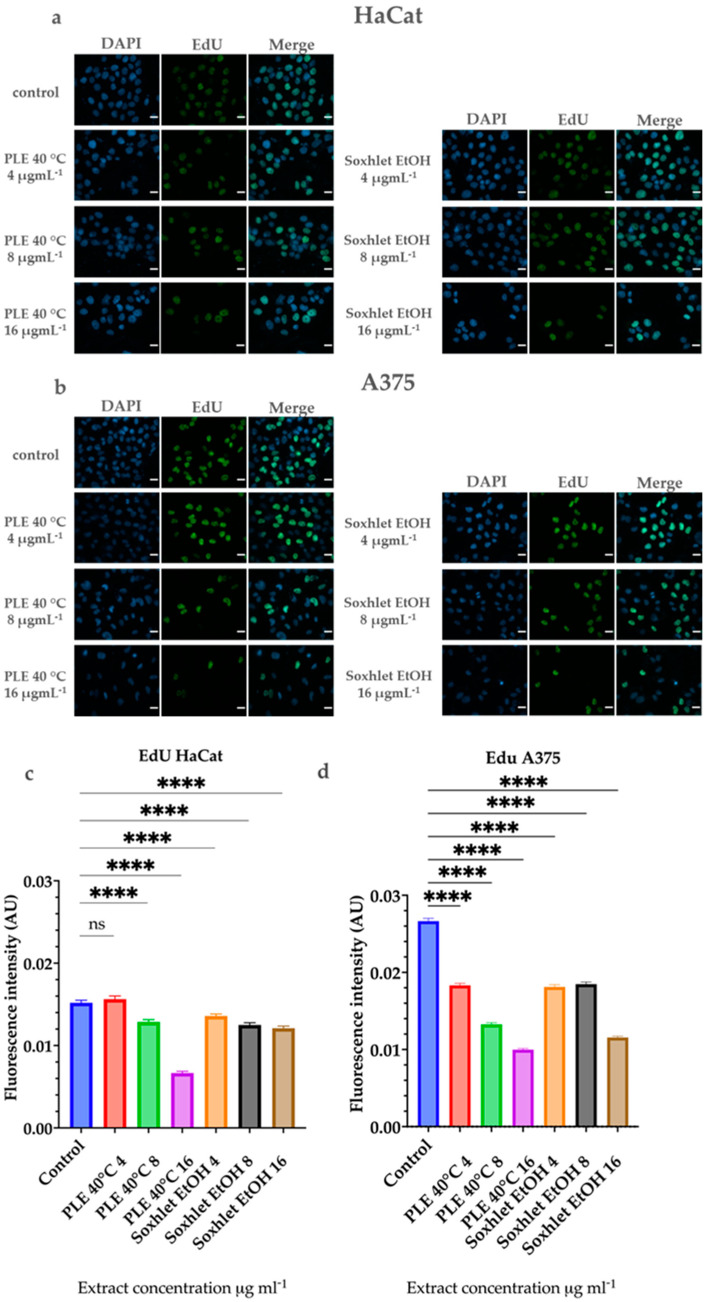
Results of the EdU cell proliferation assay after 24 h treatment with Soxhlet EtOH and PLE 40 °C extracts with concentrations ranging from 4 to 16 μgmL^−1^. (**a**,**b**) Representative images from the experiment; the mean intensity of the EdU signal (green), incorporated in newly synthesized DNA, was measured and presented as a ratio to the total DNA signal of DAPI (blue); scale bar is 20 µm. (**c**,**d**) Quantitative determination of the intensity of the EdU signal was performed using CellProfiler cell image analysis software and GraphPad Prism 10.0. Quantification is based on three independent experiments with > 5000 cells scored for each condition. The error bars represent the standard deviation of fluorescence intensity in arbitrary units (AU). Statistical analysis was performed using the ordinary one-way ANOVA with post hoc analysis using Šídák’s multiple comparisons test; **** *p* < 0.0001.

To further elucidate the cytotoxic potential of the most promising extracts and to visualize the flow cytometric analysis of the cell cycle, we performed a 5-ethynyl-2′-deoxyuridine (EdU) assay. The proliferation of human cells is determined by the fundamental process of replication that occurs in the S phase of the cell cycle. This process is comprises synthesis of a complete copy of the cell genome. If cell proliferation is suppressed, the cells either do not synthesize DNA or do not progress through the cell cycle to division. The EdU assay visualizes and quantifies cell proliferation by the incorporation of the nucleoside analog 5-ethynyl-2′-deoxyuridine into the newly synthesized DNA [40]. The results of the experiment are represented in Figure 7.

Flow cytometry-based cell cycle analysis demonstrated a reduction in the S-phase cell population with increasing treatment doses for both extracts. This observation was further confirmed by the EdU analysis. Our findings demonstrate that A375 cells exhibit a higher proliferative potential, which decreases significantly with increasing doses of the extracts. In contrast, this effect is less pronounced in HaCat cells under similar treatment conditions. Interestingly, regardless of the results for the scratch assay, the EdU assay results do not demonstrate increased proliferation of HaCat or of A375 cells with low doses. A notable increase in the EdU incorporation in newly synthesized DNA is not observed compared to the control groups, despite the lack of toxic effects and cell death. Therefore, the results of the scratch assay may be attributed mostly to increased cell motility. Previous studies in the literature suggest that plant polyphenols, and flavonoids in particular, promote skin cell migration [41,42,43].

### 2.5. Identification and Characterization of the Polyphenolic Profile of Soxhlet Ethanol and Soxhlet Water Extracts

The two Soxhlet extracts were selected for the identification and characterization of their composition for two primary reasons. First, the Soxhlet EtOH extract exhibited the highest extraction yield and showed no significant differences in total polyphenol and flavonoid composition or antioxidant activity compared to the pressurized ethanol extracts. Second, the IC_50_ value from the cytotoxicity measurement indicated that the Soxhlet ethanol extract had the best selectivity index towards A375 cells compared to HaCat cells. In contrast, the Soxhlet water extract displayed substantial differences in properties compared to both Soxhlet and PLE with EtOH. Therefore, a comparative analysis between the aqueous Soxhlet extract and the Soxhlet EtOH extract was conducted to elucidate these differences.

**Figure 8:**
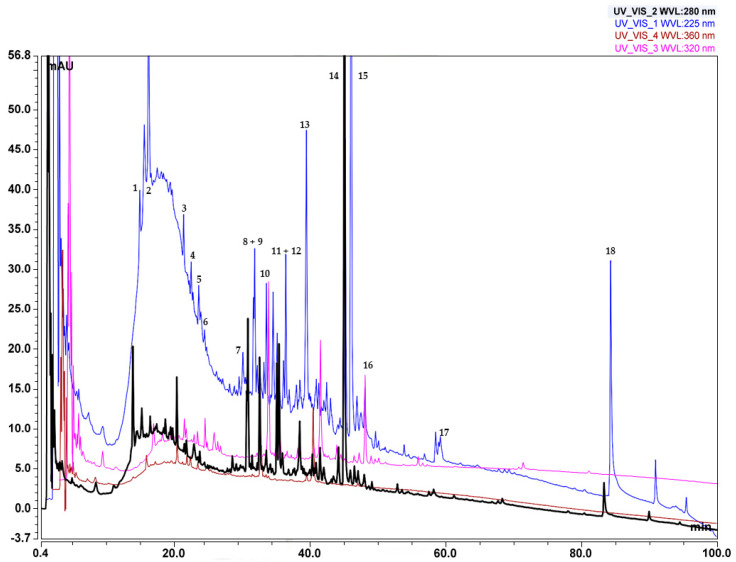
LC-MS chromatogram of *C. lechleri* twigs’ Soxhlet ethanol extract.

Compounds detected in the Soxhlet ethanol extract are shown in Table 5, numbered according to their elution order (retention times), while the LC-MS chromatogram is displayed in Figure 8. In the mobile phase with pH = 10, the retention times of some components decreased, particularly at the end of the chromatogram (Figure 8), which is an indication that those compounds have acidic properties. The substances from the fractions separated using preparative liquid chromatography facilitated the elucidation of the mechanisms of fragmentation and the structures of the main components of the extracts. Monitoring at the wavelengths chosen for the analysis can detect polyphenolic compounds, as follows: phenols and carbohydrates at λ~220–230 nm, flavonones at λ~270–290 nm, flavones at λ~320–340 nm, flavonols at λ~360–370 nm, and proanthocyanidins at λ~460–550 nm (with a secondary maximum of 270–280 nm), respectively [44]. The results displayed in Table 5 show that compounds found in the extract examined in the studied extract have an absorption maximum in these wavelength ranges. Since most of the polyphenolic compounds in plants exist in glycosylated form [45], the fragmentation spectra of the compounds in this study contain peaks, characteristic of sugar moieties. Several novel galloyl glucoside derivatives that exhibited a typical fragmentation pattern characteristic of galloyl glucose were detected in the extract. Galloyl glucose showed the molecular ion peak [M − H]^−^ *m*/*z* 331 and the fragment ions *m*/*z* 313, 271, 211, 169, and 125. The proposed fragmentation patterns of some of these derivatives, along with those of galloyl glucose, are also shown in Table 5. Fragmentation spectra of the single compounds can be found in the Supplementary Materials Section 2.5.

The compounds identified in the Soxhlet ethanol extract are derivatives of gallic acid and quercetin. The main skeleton of the gallic derivatives is galloyl glucoside.

The concentrations of galloyl glucosides are the highest in the extract. These compounds are complex and contain various groups and moieties. Some of them are synthesized by the addition of sugars, while other representatives are obtained by modification of the galloyl moiety, e.g., by methylation.

In the literature, there is ample evidence of the structure–function relationship between the galloyl modifications of flavonoids and their cytotoxic properties; see for example [46,47,48]. This relationship has been further examined by the preparation of synthetic digalloyl dimers [49].

Quercetin, on the other hand, is involved in the structure of substances identified primarily as dimethylquercetin. It is assumed that methylation occurs at the 7- and 3′- OH groups. Dimethylquercetin derivatives are mainly glucosides—at 3- and/or 7- OH groups. Galloyl modification and glycosylation have also been found to increase the cytotoxic potential of quercetin [50]. Galloylation further influences the biological properties of quercetin by enhancing its antioxidant capacity proportional to the increase in the number of hydroxyl groups [51].

It is interesting that both galloyl glucoside at -ESI and dimethylquercetin at +ESI fragment to *m*/*z* 313, which is the most abundant product ion. The differentiation between those ions with identical masses was performed by their fragmentation. Galloyl glucose showed the molecular ion peak [M − H]^−^, *m*/*z* 331, and the fragment ions *m*/*z* 313, 271, 211, 169, and 125. The [M + H]^+^, *m*/*z* 331, of (7,3′ or 3′,4′)-dimethylquercetin, also fragments to *m*/*z* 313, but the other characteristic fragments are *m*/*z* 285, 258 and 257.

Previous research has documented the occurrence of proanthocyanidins and galloylated flavonoids in the sap of *C. lechleri* [52,53]. However, to date, there is a lack of data regarding the presence of these compounds in the twigs of the species. Also, a number of the components identified in the current study have not been previously reported in the literature. Therefore, since the relevant standard substances were not available, quantification was not performed. Furthermore, it should be noted that the substances in Table 5 that are based on quercetin are also very complex and contain diverse groups and moieties that significantly affect the UV spectrum of quercetin. Hence, it is not correct to use quercetin as a reference standard for quantitative analysis.

The LC-MS chromatogram of the Soxhlet water extract is displayed in Figure S3, while the UV–vis spectra are show in Figure S4 in the Supplementary Materials.

Figure S3 Supplementary Materials shows that in comparison to the Soxhlet EtOH extract, the number of compounds in the Soxhlet water extract are considerably less. The wide variety of low molar mass substances identified in the former (Figure 8) are absent. This observation confirms the results of the TFC analysis.

At the beginning of the chromatogram, there are highly polar substances, presumed to be sugars, not retained by the hydrophobic C18 stationary phase. The other compounds present can be divided into two groups by comparing their chromatographic behavior with those of the components in Figure 8. Those belonging to the first group have molar masses of 300–500 Dal. Just a few of the substances have mass in the range 700–900 Dal, with relatively small retention times due to hydrophilicity, which is mainly a result of the glucosidic moieties in the molecules.

For the substances of the second group, it can be assumed that they have a molar mass of 900–1300 Dal. Oligomers and polymers are probably also present, but they cannot be analyzed using reversed-phase chromatography. These components have complicated UV spectra with absorption maxima *λ*~240, 300, 450, and 570 nm, which are characteristic of carbohydrates, phenols, flavones, and proanthocyanins, respectively (Supplementary Materials, Figure S4). Their peak intensities are not high due to the large molar mass of these compounds. Since glucosides absorb at *λ*~230 nm, it can be assumed that these compounds are derivatives of flavonoids with several glucoside moieties. The presence of flavonoids and proanthocyanins, though in a small amount (weak UV-maxima at *λ*~450 and 570 nm), can explain the biological activity of the water extract.

Finally, it could be also hypothesized that some of the compounds at the end of the Soxhlet water chromatogram (in analogy to the Soxhlet EtOH chromatogram) are esterified tannins (acetyl or propyl diglycosides) (Supplementary Materials, Figure S5). However, for their complete identification, more analyses are necessary, which is outside the scope of the paper.

## 3. Materials and Methods

### 3.1. C. lechleri Twigs’ Biomass

The biomass contained *C. lechleri* twigs cut into pieces 3–4 cm long, with a diameter of 1–2 cm, and purchased from a local herb pharmacy in Aguascalientes, Mexico.

### 3.2. Chemicals and Reagents

For the Soxhlet, scCO_2_, and extraction with pressurized ethanol, the following chemicals were used: *n*-hexane ≥ 99% and ethanol ≥ 99.8% purchased from Honeywell Riedel-de-Haen (Seelze, Germany); bone dry grade CO_2_ (99.99% pure; no water, Messer, Sofia, Bulgaria).

For the TPC and antioxidant activity assays, the following reagents were used: 2,2-diphenyl-1-picrylhydrazyl (DPPH) (Sigma-Aldrich, St. Louis, MO, USA); methanol ≥ 99.9% purchased from Honeywell Riedel-de-Haen (Seelze, Germany); Folin–Ciocâlteu reagent 2 N, sodium carbonate (Merck, Darmstadt, Germany), and gallic acid (Sigma-Aldrich, St. Louis, MO, USA).

For the TFC assay, sodium nitrite (NaNO_2_) (Sigma-Aldrich, St. Louis, MO, USA), aluminum chloride hexahydrate (AlCl_3._6H_2_O) (Sigma-Aldrich, St. Louis, MO, USA), sodium hydroxide (NaOH) (Sigma-Aldrich, St. Louis, MO, USA), and rutin trihydrate (Sigma-Aldrich, St. Louis, MO, USA) were used.

Cell lines and media: HaCat cells (product number 300493) were purchased from CLS Cell Lines Service GmbH, Eppelheim, Germany; A375 cells (product number CRL-1619) were purchased from ATCC (Manassas, WV, USA); Dulbecco’s Modified Eagle’s Medium (DMEM) was purchased from Thermo-Fischer Scientific (Waltham, MA, USA); trypan blue stain (0.4%), MTT (3-(4,5-dimethylthiazol-2-yl)-2,5-diphenyltetrazolium bromide), trypsin-EDTA (0.5%), streptomycin, and penicillin were purchased from Thermo-Fischer Scientific (Waltham, MA, USA).

Microscopic analysis: Phosphate buffered saline (PBS) was purchased from Thermo Fisher Scientific Inc. (Waltham, MA, USA); Triton™ X-100, TWEEN^®^ 20, Trizma^®^ base, and bovine serum albumin (BSA) were purchased from Sigma-Aldrich (St. Louis, MO, USA); coverslips were purchased from Epredia (Kalamazoo, MI, USA).

Analytical methods: Ethyl acetate, p.a., ethyl ether, p.a., methanol, p.a., formic acid, FA (HPLC grade). Solvents: MeOH, ACN, and water (HPLC gradient grade) were purchased from Merck (Darmstadt, Germany).

### 3.3. Extract Recovery

#### 3.3.1. Preliminary Preparation of the Material

In order to fit into a blade mill, *C. lechleri* twigs were initially crushed with a hammer. The biomass was then ground into the mill (Ika, Werke, M20) for 30 min at 10 min intervals in order not to overheat both the mill and the biomass. Consequently, the crushed biomass was subjected to two 15 min cycles at 500 rpm in an agate ball mill (Retsch, model S100). Finally, the biomass was sifted through a 0.40 mm sieve, and only this fraction was used in the experiments.

To analyze the biomass particle size, the Malvern-Mastersizer 3000 equipment(Malvern Panalytical-Spectris, Worcestershire, United Kingdom), available at Pharmalab’s laboratory, ISEL, was used. The Mastersizer 3000 works by laser diffraction (LSD), i.e., measuring the intensity of the scattered light when a laser beam passes through a sample of scattered particles. Thus, accurate particle size distributions for both wet and dry dispersions (particle size range 10–3.5 nm) are provided.

These data are then analyzed to calculate the size of the particles that created the scattering pattern. The main parameters provided by the measurements in the equipment are D(10), D(50), and D(90), where D(10) is the diameter that has 10% of the total volume of the particles in the system, D(50) is the diameter that divides the distribution in half, i.e., above and below this value is 50% of the total volume of the particles, and D(90) is the diameter that has 90% of the total volume of the particles in the system. It was determined that the last 3 parameters give a mean particle size of 0.236 mm (Table 6).

The moisture content was measured using a thermogravimetric process in an electronic balance (Kern MRS 120-3) with the “Standard” heating program at a temperature of 104.85 °C until a constant weight was achieved. Three replicas were conducted, and the resulting value was (2.09 ± 0.16)%.

#### 3.3.2. Soxhlet Extraction

One- and two-step extractions were performed with solvents with different polarities. In the one-step Soxhlet extractions ethanol, and water were used, while in the two-step extraction, *n*-hexane was initially applied, followed by ethanol recovery of the residual biomass. The extractions were conducted using the protocol reported in detail in our previous work [54]. In brief, the Soxhlet apparatus used was ISOLAB NS29/32 + 34/35 (Merck KGaA, Darmstadt, Germany). In all experiments, the extraction cartridge was filled with 5.0 ± 0.1 g of the biomass, the solvent volume was 150 mL, and the ratio of solvent to solid was 30 to 1. All extractions were performed for three hours. In the two-step extraction, *n*-hexane extraction was first used. Subsequently, the residual matrix was subjected to extraction with ethanol. Hei-VAP Rotary Evaporator (Heidolph Instruments GmbH&Co. KG, Schwabach, Germany) was employed for the evaporation of the solvent from the liquid extract. Next, the dry extracts obtained were additionally dried at 40 ± 2.0 °C to a constant weight in an air circulation oven. Then, they were placed in glass vials and kept at 4 °C for further analysis. All experiments were conducted in triplicate, and total extraction yield was expressed as mean ± standard deviation.

#### 3.3.3. Supercritical Fluid Extraction (SFE)

*C. lechleri* twig biomass was extracted with neat scCO_2_ and with scCO_2_ + 10% ethanol in a flow apparatus (SFT-110-XW, Supercritical Fluid Technologies Inc., Newark, DE, USA). The CO_2_ pressure was maintained by an SFT Nex10 SCF pump actuate from the compressor model HYAC50-25 (Hyundai, Republic of Korea). An additional pump (LL-Class, USA) from SFT (Inc., Newark, DE, USA) was used in experiments with a co-solvent. A detailed description of the equipment is given in our previous works [55]. About 5 g of dry biomass sample was placed in the processing vessel. The solvent was removed from the liquid extracts through vacuum evaporation on the Hei-VAP Rotary Evaporator (Heidolph Instruments GmbH&Co. KG, Schwabach, Germany). Subsequently, the extracts were further dried at 40 ± 2.0 °C to a constant weight in an air circulation oven.

#### 3.3.4. Extraction with Pressurized Ethanol

For the experiments with the pressurized ethanol, the same apparatus as in the case of SFE (SFT-110-XW, Supercritical Fluid Technologies Inc., Newark, DE, USA) was employed. The solvent was pressurized with HPLC pump Alltech, model 426 (Germany). The experiments were carried out with 5 g of dried sample at 200 bar and two temperatures (40 and 60 °C), and the ethanol flow rate was 1.5 mL·min^−1^.

A rotary evaporator (Heidolph Instruments GmbH&Co. KG, Schwabach, Germany) was used for the recovery of extracts, which were afterwards further dried to a constant weight in an air circulation oven at 40 ± 2.0 °C.

### 3.4. Total Phenolic Content, Antioxidant Activity, and Total Flavonoid Content (TFC)

The TPC was determined using the Folin–Ciocâlteu method [56]. The treated samples of the extracts acquired coloring proportional to their TPC. They were analyzed using a double-beam UV–vis spectrophotometer (UNICAM ^®^-Helios β) at 765 nm and compared to a calibration line obtained with gallic acid (GA) as a control reference.

The antioxidant activity was determined using the DPPH method, which is based on the neutralization of free radicals emitted by the DPPH solution [57]. The samples were analyzed spectrophotometrically at 517 nm.

The IC50 value that is derived from the DPPH tests represents the capacity of the extract to inhibit free radicals and is calculated according to

(2) $$\mathrm{IC}\;=\frac{\mathrm{Ao}\;-\;\mathrm{As}}{\mathrm{Ao}}\times 100\;$$

where Ao is the absorption of a control sample (methanol) and As is the absorption of an extract sample.

The aluminum chloride colorimetric assay was used for the determination of the TFC in four of the red extracts. The protocol applied is the one described in [58], with slight modifications. Briefly, 1 mL aliquot of the extracts in appropriate dilution was added to a 10 mL volumetric flask containing 4 mL of distilled water. First, 0.3 mL 5% NaNO_2_ was added to the flask. After 5 min, it was followed by the addition of 0.3 mL 10% AlCl_3_.6H_2_O. One minute later, 2 mL of 1 M NaOH was added to the flask, and immediately after that, the final volume of the reagent mixture was filled up to 10 mL with distilled water. Absorbance was measured at 510 nm with Eppendorf BioSpectrometer ^®^ (Eppendorf SE, Hamburg, Germany), and a mixture of all reagents except for extract samples was used for a blank. A calibration curve was built using rutin trihydrate as a standard, and the results were expressed as mg of rutin equivalents (RE) per gram of dry extract (mg·de^−1^) (Supplementary Materials, Section 3.4).

### 3.5. Cell Line Cultivation

Human keratinocytes HaCat and human malignant melanoma cells A375 were cultured in an incubator under a humidified atmosphere at 37 °C and with 5% gaseous CO_2_. The cell culture media for both cell lines consisted of Dulbecco’s Modified Eagle’s Medium (DMEM) supplemented with heat-inactivated fetal bovine serum to achieve a final concentration of 10% (*v*/*v*), along with a combination of penicillin (final concentration of 100 IUmL^−1^) and streptomycin (100 mg·mL^−1^).

### 3.6. Cytotoxicity Measurement

The MTT cytotoxicity assay [59] was used for cytotoxicity screening of the chosen extracts. HaCat cells were grown until confluency for 24 h in 96-well plates at a seeding density of 1 × 10^4^ cells per well. A375 cells were grown until confluency for 24 h in 96-well plates at a seeding density of 4 × 10^3^ cells per well. Thereafter, treatment with the extracts was performed in the concentration range between 4 µg·mL^−1^ and 512 µg·mL^−1^ with two-fold serial dilutions for both cell lines. The stock solution of the Soxhlet water extract was prepared with distilled water at a concentration of 10 mgmL^−1^, while the stock solutions of the Soxhlet EtOH and PLE extracts were prepared with DMSO at a concentration of 50 mgmL^−1^ so that the final dilution for the treatment did not contain more than 1% DMSO. All treatment dilutions were prepared in DMEM. After 72 h incubation with the extracts, the medium was removed and replaced with a phenol-free medium with 0.5 mgmL^−1^ MTT. The cell culture plates were incubated for 2.5 h at 37 °C, and after that, the MTT-containing medium was removed. The resulting formazan crystals were solubilized with 100 μL DMSO per well. Absorbance was measured on a Varioscan Lux multifunctional microplate reader (Thermo Fisher Scientific, Waltham, MA, USA) at 550 nm. Dose–response curves and IC50 values were obtained using GraphPad Prism 10 software.

### 3.7. Wound Healing Assay

Human normal keratinocytes HaCat cells or A375 epithelial cells derived from malignant melanoma were seeded in 12-well plates at a density of 5 × 10^4^ cells per well until they formed confluent monolayers. To create uniform wounds, a 20 µL pipette tip was used. Following a medium wash to eliminate cellular debris, the injured cell layers were exposed to a medium containing 2% FBS to restrict cell proliferation. Subsequently, they were treated with final concentrations of 4, 8, or 16 μgmL^−1^ of Soxhlet H_2_O, ethanol, PLE 40 °C extracts, or PBS as a control. The progress of wound healing was monitored by capturing images at specific time points (0, 4, and 24 h), and the extent of the cell-free wound areas was quantified using Fiji (ImageJ software, version 2.1.0). Five separate images were taken for each experimental condition at any time point. The data were derived from two separate trials. Statistical analysis was performed using GraphPad Prism 10 software.

### 3.8. Trypan Blue Exclusion

To determine the percentage of dead cells following the treatment with Soxhlet EtOH and PLE 40 °C extracts, the trypan blue exclusion assay was used. A375 and HaCat cells were plated in 12-well plates at a seeding density of 2 × 10^5^ cells per well and treated for 24 h with the extracts in the concentration range between 4 and 64 μgmL^−1^ with two-fold serial dilutions. The cells were harvested with trypsin-EDTA solution in PBS, and 10 µL of the cell suspension was mixed with 0.4% trypan blue stain (Invitrogen) in PBS in a 1:1 ratio. After that, the percentage of dead cells was assessed using Invitrogen™ Countess™ 3 Automated Cell Counter (Invitrogen, Waltham, MA, USA). The cell images were taken with a Zeiss Axiovert 200M fluorescence inverted microscope using the Plan-Apochromat 10×/0.45 M27 objective. Statistical analysis of the results was performed using GraphPad Prism 10 software.

### 3.9. Annexin V Staining Assay

To determine the percentage of apoptotic and necrotic cells after treatment with the Soxhlet EtOH and PLE 40 °C extracts, the Annexin V Apoptosis Detection Kit FITC/PI (Thermo Fisher Scientific, Waltham, MA, USA) was used, following the manufacturer’s instructions. Briefly, A375 and HaCat cells were plated in 12-well plates at a seeding density of 2 × 10^5^ cells per well and treated for 24 h with the extracts in the concentration range between 4 and 32 µg·mL^−1^ with two-fold serial dilutions. After harvesting the cells from the plates, they were resuspended in 100 µL Annexin V binding buffer (AVBB). After that, the cell suspension was incubated with 5 µL of Annexin V-FITC for 15 min. This was followed by washing with AVBB, subsequent resuspension in 200 µL AVBB containing PI, and incubation on ice in the dark for 30 min. The labeled cell suspensions were analyzed using flow cytometry on a Becton Dickinson FACScalibur instrument (BD Biosciences, San Jose, CA, USA). The percentages of live, early apoptotic, late apoptotic, and necrotic cells were quantified using FlowJo v.10.8.1 software (BD Biosciences, Ashland, OR, USA).

### 3.10. Cell Cycle Analysis

For analysis of the cell cycle progression after treatment with the Soxhlet EtOH and PLE 40 °C extracts, propidium iodide (PI) staining was used. Both cell lines were plated in 6-well plates at a seeding density of 5 × 10^5^ cells per well and treated for 24 h with the extracts in the concentration range between 4 and 16 µg·mL^−1^ with two-fold serial dilutions. After the treatment, the cells were harvested, washed with PBS with centrifugation at 200× *g* for 5 min at 4 °C, and fixed with 70% ethanol at 20 °C overnight. After that, the cell pellet was washed with cold PBS, centrifuged at 200× *g* for 10 min at 4 °C, and resuspended in 500 µL PI 0.1% Triton X-100 solution in PBS with 25 µg·mL^−1^ PI, containing 200 µg·mL^−1^ DNAse-free RNAse A (Sigma-Aldrich, St. Louis, MO, USA). After incubation for 30 min at room temperature in the dark, analysis was performed using flow cytometry on a Becton Dickinson FACScalibur instrument (BD Biosciences, San Jose, CA, USA). The percentages of G1, S, and G2-phase cells were quantified using FlowJo v.10.8.1 software (BD Biosciences, Ashland, OR, USA).

### 3.11. Ethynyl-2′-Deoxyuridine (EdU) Labeling

HaCat and A375 cells were cultured on ⌀ 12 mm coverslips at a seeding density of 2.5 × 10^4^ cells per coverslip and grown for 48 h, until they reached 70% confluency. After that, the cells were treated with concentrations of 4, 8, and 16 μgmL^−1^ of Soxhlet EtOH and PLE 40 °C extracts for 24 h, washed with 1 × PBS, and incubated with 25 µM EdU for 30 min. Then, a fixation step with methanol for 10 min at 20 °C followed. The fixed cells were washed with 100 mM Tris-HCl (pH 7.2) for 5 min prior to permeabilization with 0.1% Triton X-100 in PBS for 5 min. After subsequent washing with 1 × PBS for 5 min, the cells were incubated in blocking buffer with 3% BSA and 0.1% Tween-20 in 1 × PBS for 45 min at room temperature. Immediately after that, a “click” reaction was carried out in a reaction mixture containing 100 mM Tris-HCl, pH 7.6, 4 mM CuSO_4_, 10 µM Alexa Fluor 488 azide, and 100 mM sodium ascorbate for 30 min at room temperature. The coverslips were washed with EtOH and distilled water and mounted with ProLong™ Gold Antifade (Invitrogen), containing 0.5 µg·mL^−1^ DAPI (Cell Signaling Technology, Danvers, MA, USA), for counterstaining of the cell nuclei. The cell images were taken with a Zeiss Axiovert 200M fluorescence inverted microscope (Zeiss, Germany) using the Plan-Apochromat 63×/1.4 Oil DIC M27 objective. The imaging data were analyzed using CellProfiler software, version 4.2.6 [60].

### 3.12. Statistical Analysis

The results represent the mean of two or three independent experiments performed in duplicate or quadruplicate. The results were expressed as mean value ± standard deviation (SD) or standard error of the mean (SEM). Statistical analysis was performed using ordinary one-way ANOVA or two-way ANOVA with post hoc analysis using Tukey’s multiple comparison test. The statistical significance was defined as *p* < 0.05. Data analysis was carried out using GraphPad Prism (version 10.0).

### 3.13. Characterization of the Soxhlet Ethanol and Soxhlet Water Extracts

The composition of the extracts was determined using the UHPLC system UltiMate 3000 with photodiode array scanning in the UV range 200–650 nm and the Thermo Fisher Orbitrap Elite Mass Spectrometer (Thermo Fisher Scientific, Waltham, MA, USA).

#### 3.13.1. Preparative Techniques

##### Column Chromatography (CC)

Both Soxhlet ethanol and Soxhlet water extracts were subjected to column chromatography in order to purify the compounds depending on their polarity or hydrophobicity. The methodology will be outlined in the example of the Soxhlet ethanol extract, which, in what follows, will be referred to as the extract:

A sample of 0.5 g of dried extract was dissolved in 50 mL distilled water with vortex. The nonpolar and weakly polar components of this solution were extracted three times with 40 mL ethyl acetate (EtOAc), and the organic phases were combined and evaporated at 40 °C with a low-pressure evaporator.

The dried residue was dissolved again in 10 mL distilled water and loaded onto a 50 × 4 cm column with amberlite XAD7-HP resin.

The compounds of the sample were eluted with 5 column volumes of distilled water. Nonpolar and weakly polar compounds were cleaned from sugars and salts by discarding the first column volume water (eluent). The brown zone at the top of the column was removed with 3 column volumes of methanol. The eluates were evaporated at 40 °C with a low-pressure evaporator.

The dry residues of both eluates of the column separation were reconstituted with 5% MeOH in water, separated using preparative LC, and then analyzed using UHPLC-DAD and LC-MS [61].

As a result, the compounds of the dry extracts were divided as (a) nonpolar and weakly polar (in the EtOAc phase) and (b) polar (in the water phase).

The weight of the dry residue of the aqueous eluate of the EtOAc phase, a, was 54 mg, and that of the methanol eluate of the phase was 13 mg.

The aqueous phase was also separated using column chromatography.

The dry weight of the aqueous eluate of the water phase, b, was 9 mg, and the dry weight of the methanol eluate (brown zone) was 90 mg. Thus, it was found that the water-soluble nonpolar compounds in the extract were 12.6%, and the polar substances 20.6%. It is assumed that the rest of the dry methanolic extract—334 mg (66.8%), contains high molecular substances, such as lignins, cellulose, etc., which are not soluble in water at room temperature.

##### HPLC Analysis

The reconstituted dry residues of the fractions of the column chromatography were analyzed using Thermo Fisher Ultimate 3000 LC system (Thermo Fisher Scientific, Waltham, MA, USA) consisting of a degasser, automatic injector, column thermostat, and diode array detector.

##### UV Detection

The UV absorption was monitored at five wavelengths: λ~220–230 nm; λ~270–290 nm, λ~320–340 nm, λ~360–370 nm, and λ~460–550 nm.

Full UV spectra were obtained using a DAD 3D profile.

##### Preparative HPLC

For a more reliable identification of the components of the eluated fraction, the substances in trace amounts and contaminants from the matrix effect of the column chromatography were removed using preparative liquid chromatography.

An aliquot of about 50 mg of the purified and dried methanol and water eluates was dissolved with 0.5 mL of mobile phase A, and 150 µL samples were injected into a 250 × 10 mm preparative column packed with Kinetex 5 micron EVO C8 100A (Phenomenex, part No 00G-4633-HO. The chromatographic conditions were the same as those reported for the separation with the analytical column (next section), but the flow rate was 3 mL/min. Compounds were collected every 15 min in six fractions.

#### 3.13.2. Analytical RP HPLC

The compounds of the column fraction were separated using Kinetex 2.6 um EVO C18 100 LC column 150 × 2.1 mm, 2.6 micron (part No OOF-425-AN, Phenomenex).

Mobile phase: A—Water with 0.2% NH_4_OH (pH = 10), B—ACN with 0.2% NH_4_OH; flow rate −0.2 mL·min^−1^.

Gradient elution: 1. Slow gradient for separation of 0′(2% B)—5′(2%B)—95′(100%B)—105′(95%B)—106′(2%B); 2. Fast gradient for the separation of a limited number of components, e.g., the fractions from the preparative TH. The initial and final concentrations of the organic modifier, ACN, and the slope of the gradient were determined by the properties of the components.

##### LC-MS

The compounds of the reconstituted dried eluates and the collected compounds of the fractions of the preparative LC were separated under the same conditions as those for the analytical column.

Electrospray ionization (ESI) was performed in both the negative and positive ion modes. The parameters for analysis were set as follows: capillary voltage 3 kV at negative ion mode and 3.5 kV at positive ion mode, cone gas flow 50 L·h^−1^, desolvation temperature 280 °C, and desolvation gas flow 900 L·h^−1^. Mass spectra were detected in the ESI between *m*/*z* 100 and 1200 atomic mass units. Chemical constituents were identified based on their fragmentation patterns.

Two types of mobile phases were applied—with pH = 3 and pH = 10.

The compounds were tentatively identified by comparing their retention time (tR), mass spectra, and UV spectra.

## 4. Conclusions

The present study outlined the successful application of various extraction techniques to recover bioactive compounds from *C. lechleri* twigs, focusing on advanced, environmentally friendly methods. The extracts demonstrated significant cytotoxic activity, particularly against malignant melanoma cells, and exhibited promising antioxidant and wound-healing properties.

The findings of this investigation offer a new perspective for the sustainable transformation of *C. lechleri* twig biomass, which is often regarded as waste, into valuable phytochemicals for various industries, including pharmaceuticals, nutraceuticals, and cosmetics. Future research should explore the molecular mechanisms underlying these bioactivities and consider further validation of species identification using molecular markers, which will help unveil the full potential of *C. lechleri* twigs.

## Figures and Tables

**Table 1:** Influence of the solvent in Soxhlet extraction on the yield of *C. lechleri* twigs.

Extraction Method	Extraction Conditions	Extraction Yield (wt %)	Color of the Extracts
Soxhlet extraction with water	100 °C, 3 h	7.54 ± 0.4	red
Soxhlet extraction with EtOH	78 °C, 3 h	16.07 ± 0.94	red
First-step Soxhlet extraction with *n*-hexane	68 °C, 3 h	2.38 ± 0.16	green
Second-step Soxhlet extraction with EtOH	78 °C, 3 h	11.61 ± 0.5	red

Extraction yield expressed in wt % (mean ± standard deviation).

**Table 2:** Influence of the different extraction methods on the TPC and IC 50%.

Extraction Method	Extraction Conditions	TPC, mgGAE·g_de_^−1^	IC 50, mg_de_·L^−1^
Soxhlet extraction with water	100 °C, 3 h	133.18 ± 3.01	145.83
Soxhlet extraction with EtOH	78 °C, 3 h	223.85 ± 3.68	158.41
Soxhlet extraction with *n*-hexane (first step)	68 °C, 3 h	3.47 ± 0.09	14,826.7
Soxhlet extraction with EtOH (second step)	78 °C, 3 h	220.86 ± 3.0	164.52
PLE with EtOH	200 bar, 40 °C	248.78 ± 4.10	135.17
	200 bar, 60 °C	199.92 ± 2.58	170.36
SFE with neat CO_2_	500 bar, 60 °C	26.56 ± 1.99	3108.40
SFE with CO_2_ +10%EtOH	200 bar, 40 °C	26.55 ± 0.93	2802.318
	200 bar, 60 °C	27.42 ± 1.19	3000.305
	300 bar, 40 °C	29.8 ± 1.47	1842.585
	300 bar, 60 °C	28.38 ± 1.16	1783.39
	500 bar, 60 °C	31.88 ± 1.80	2794.54

Relative standard deviation (RSD): RSD_IC50_ = ±1.56%.

**Table 3:** Total flavonoid content of the red extracts.

Extraction Method	Extraction Conditions	TFC, mgRE·g_de_^−1^
Soxhlet extraction with water	100 °C, 3 h	33.4 ± 1.91
Soxhlet extraction with EtOH	78 °C, 3 h	89.83 ± 2.38
PLE with EtOH	200 bar, 40 °C	129.11 ± 3.12
	200 bar, 60 °C	100.97 ± 2.69

**Table 4:** Cytotoxicity of Soxhlet EtOH, Soxhlet H_2_O, and PLE with EtOH 40 °C and 60 °C extracts was determined using the MTT-dye reduction assay after 72 h of continuous exposure to HaCat and A375 cell lines. GraphPad Prism 10 software was used for the calculation of half-inhibitory concentrations IC_50_, presented here in μg·mL^−1^. The selectivity index (SI) was calculated based on the IC_50_ values obtained using MTT analysis, as the ratio IC_50_ HaCat/IC_50_ A375.

Cell Line/Extract	Soxhlet EtOH	Soxhlet H_2_O	PLE 40 °C	PLE 60 °C
HaCat	63.47 ± 3.01	249.8 ± 22.21	33.98 ± 2.02	30.50 ± 1.74
A375	13.31 ± 1.07	77.97 ± 3.43	18.91 ± 1.11	17.38 ± 1.29
SI	4.77	3.20	1.80	1.75

**Table 5:** Tentative characterization of phenolic compounds in C. lechleri twigs’ Soxhlet ethanol extract using LC-MS.

No	t_R_ (min)	UV (nm)			Tentative Identification	Molecular Weight (*m*/*z*)	[M + H]^+^/[M − H]^−^ (*m*/*z*)	Fragments (*m*/*z*)
		λ_1_	λ_2_	λ_3_				
1	13.93	220 m	285		7,3-O-dimethyl-Quarcetin-3-O-ethylacetate	391.254	M−: 391.254	358.25; 329; 313.17 284.17; 238.09; 163.08
2	14.96	225 m	275		Pyrogallol-O-methyl-Galloyl-Glucoside	454.343	M−: 453.173	313.17; 284.17; 163.08
3	19.20	235 m	300	350	7,3′-O-dimethyl-Quercetin	330.2368	M−: 329. 236	311.24; 293.23 283.23; 227.23
4	21.14	215 m	320	360	7-O-Glc-3′-O-CH3-3-O-Acetyl-Quercetin	519.2632	M−: 520.266	358.14; 313.09 284.17; 239.09
5	21.45	216 m	280	403	7-O-Rha–Rha′-3′-O-Methyl-Quercerin-3-O-propionatate	694.173	M−: 693.172	536.09; 391.17 358.09; 313.09 284.09
6	21.86	215 m	320	360	7,3′-O-CH3-3-O-Acetyl-Quercetin	370.0934	M+: 371.0931	313.09; 285.09 214.34
7	29.21	214	236	353 m	7,3′-O-dimethyl-Quecetin-3-O-Glc	492.4312	M+: 491.344	329.09; 311.09 293. 09
8	30.23	220 m	288	355	7-O-Glu-3′,5′-O-Dimethyl-1-0-Acetyl-Quercetin	535.174	M+: 536.264	374.17; 311.17 284.17; 239.17
9	30.32	216 m	286	340	7-O-Rha-3′-O-Methyl-1-0-Methyl-Quercetin	535.174	M−: 536.172	391.25; 371.09 329.17; 313.17 284.17; 239.09
10	32.10	216 m	279	325	7-O-3′-O-Methyl-3-Acetyl-Quercetin	372.0945	M−: 371.173	329.09; 313.17 284.09; 239.09
11	33.14	216 m	279	326	7-O-Glu-3′-O-Methyl-3-acetyl-Quercetin	537.0945	M−: 536.0932	374.17; 329.09 313.01; 284.09
12	33.25	216 m	286	340	7-O-Glu-3′-O-Methyl-1-acetyl-Quercetin (isomer of comp. No 11)	537.0945	M−: 536.0932	374.17; 329.09 311.09; 284.09
13	38.50	212 m	324	443	7-O-Glu-3′-O-Methyl-1-Acetyl-Quercetin	537.0945	M+: 536.0932	374.17; 329.09 313.09; 235.09 163.08
14	44.10	218 m	276	349	7,3′-dimethyl-3-O-Acetyl-Quercetin	375.1756	M+: 376.1744	315.09; 285.09 230.09
15	45.26	216 m	230	280	4-Ethyl-Galloyl-Glucoside	359.1723	M−: 358.1714	330.17; 313.09 295.09; 267.09 125.08
16	45.95	212 m	286	326	7-O-Glu-3′,5-O-Dimethyl-1-Acetyl-Flavanol	537.0945	M−: 536.092	374.17; 329.09 311.09; 284.09
17	58.10	218 m	276	349	7,3′-dimethyl-3-O-Acetyl-Quercetin	377.1756	M−: 376.174	315.09; 285.09 230.0
18	68.22	275 m	320	365	3′-O-methyl-Quarcetin-3-O-Rha-72-O-3′2,5′2-dimethylQuarcetin-3-O-ethylacetate	853.431	M−: 852.430	536.17; 391.25 358.17 329.09; 313.09 284.09; 261.09
19	82.40	219 m	288		Rha-7-O-3′,5′-dimethylQuercetin-3-O-ethylacetate	685.264	M−: 684.262	536.26; 391.34 358.25; 313.17 284.17; 261.09
20	89.82	229 m	300	440	(Oligo)Flavonoids-Glucosides (Lignins)			
21	93.90	230 m	240	300 440	(Oligo)Galloyl-Glucosides (Lignins)			
22	105.5	233 m	240	320 440	(Oligo)Galloyl-Glucosides (Lignins)			

Rha—rhamnose (146); Glc—glucose (162); _m_—λ_max_; [M + H]^+^ = [M + 1]^+^; [M − H]^−^ = M^−^

**Table 6:** Particle sizes determined using LSD.

	Dx (10) (μm)	Dx (50) (μm)	Dx (90) (μm)
Mean	10.9	236	810
1xStd Dev	1.97	60.7	127
1xRSD (%)	18.1	25.7	15.7

## Data Availability

Data are contained within the article.

## Supplementary Materials

The following supporting information can be downloaded at https://www.mdpi.com/article/10.3390/molecules29174161/s1. Figure S1: Dose–response curves from the MTT assay for cytotoxicity of the Soxhlet and PLE extracts. HaCat cells (a) and A375 cells (b) were treated for 72 h with concentrations ranging from 4 to 512 µgmL^−1^. Data are presented as the mean of three independent experiments; Figure S2: Microscopic images from the morphological analysis of cell shape alterations of HaCat cells after 6 h (a) and 24 h (b) and A375 cells after 6 h (c) and 24 h (d) when treated with Soxhlet EtOH and PLE 40 °C extracts; concentrations of the treatment range from 4 to 64 µgmL^−1^; scale bar is 100 µm; mass spectra of the tentative identified compounds; Figure S3: Chromatogram of *C. lechleri* twigs’ Soxhlet water extract; Figure S4: UV–vis spectra of compounds from *C. lechleri* twigs’ Soxhlet water extract; Figure S5: MS spectrum of peak with tR 34 and 43 min—oligomers and polymers (molar mass ranging from 500 to 3000); Figure S6: Calibration curve for quantification of the total flavonoid content in the *C. lechleri* extracts.
