# Supplementary material for: Novel Insights into the Biological Activity of Croton lechleri Twigs Extracts and Advancements in Their Sustainable Recovery
Source: Molecules. 2024 Sep 2;29(17):4161. doi: 10.3390/molecules29174161 (PMC11397310; doi:10.3390/molecules29174161)
Supplement: Supplementary file 1 [file molecules-29-04161-s001.zip › molecules-3134300-supplementary.pdf]

Supplementary materials

Section 2.4 – Bioactivity results

2.4.1. Cytotoxicity

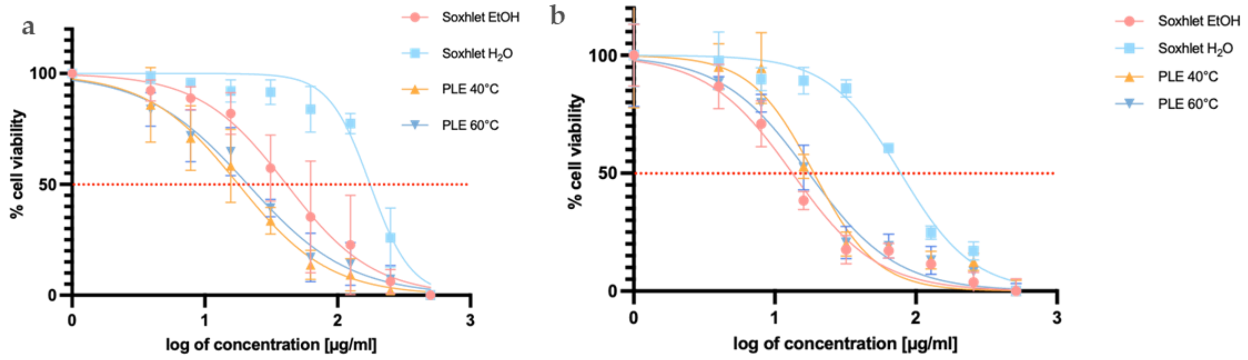

Figure S1 – Dose-response curves from the MTT assay for cytotoxicity of the Soxhlet and PLE extracts. HaCat cells (a) and A375 cells (b) were treated for 72 h with concentrations ranging from 4 to 512 μgmL^{-1}. Data is presented as the mean of three independent experiments.

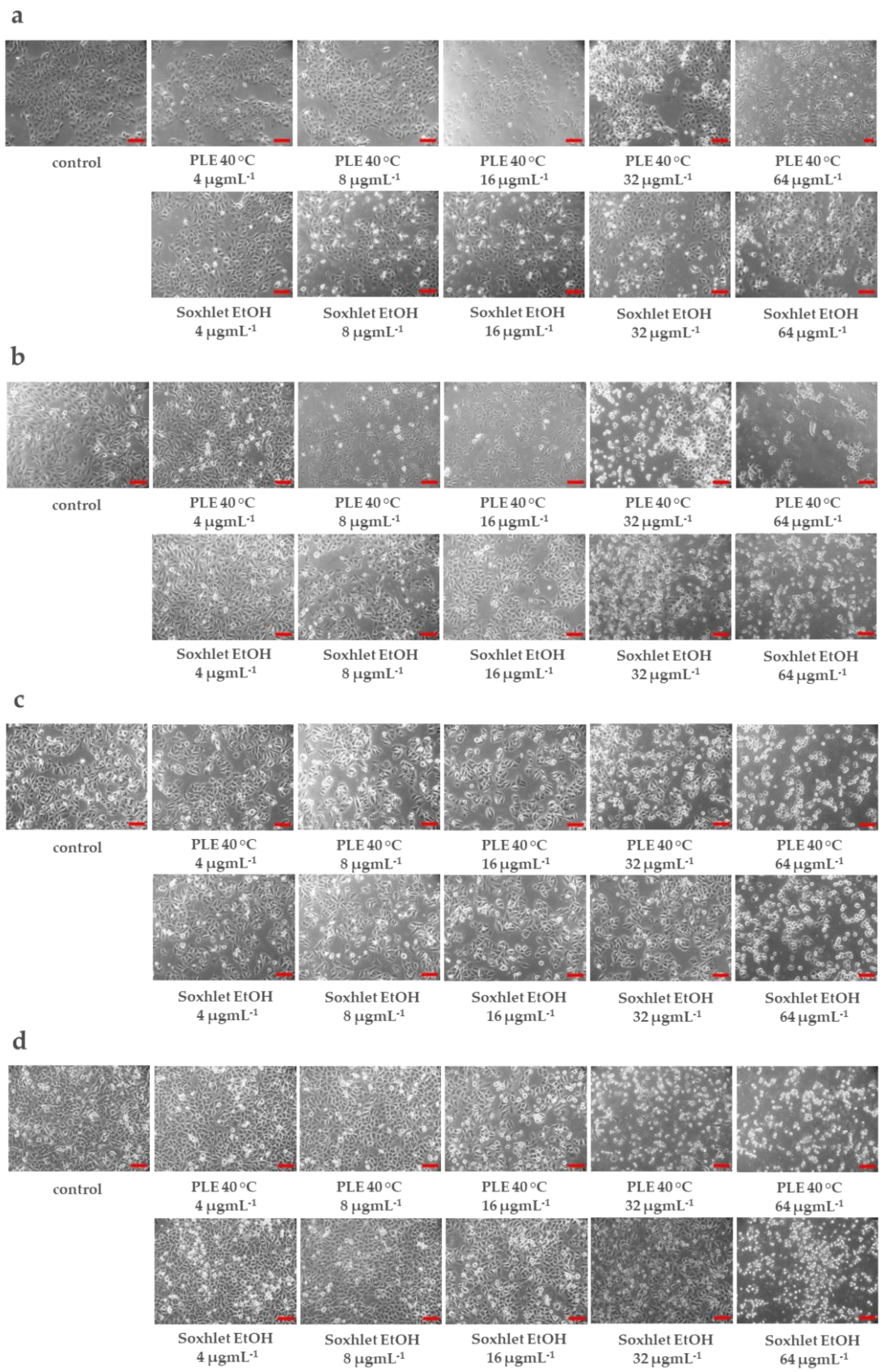

Figure S2 – Microscopic images from the morphological analysis of cell shape alterations of HaCat cells after 6 h (a) and 24 h (b) and A375 cells after 6 h (c) and 24 h (d), when treated with Soxhlet EtOH and PLE 40 °C extracts; concentrations of the treatment are ranging from 4 to 64 μgml^{-1} **;** Scale bar is 100 μm.

Section 2.5 - Identification and characterization of the polyphenolic profile of Soxhlet ethanol and Soxhlet water extracts

Mass spectra of the tentative identified compounds

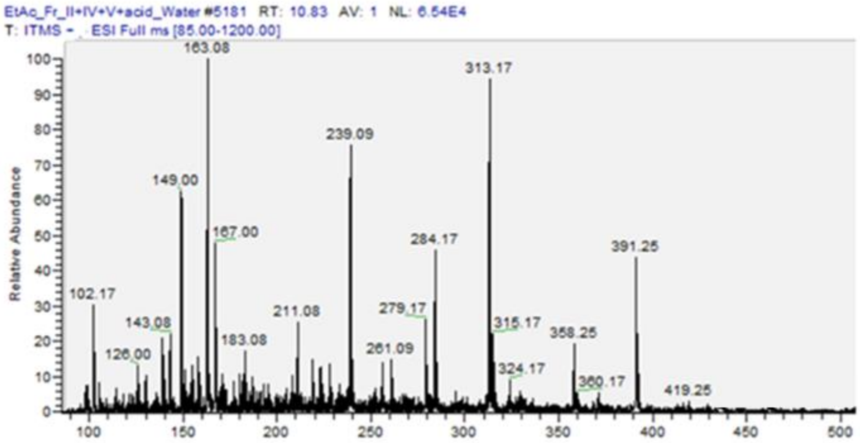

1: 7,3-O-dimethylQuarcetin-3-O-ethylacetate

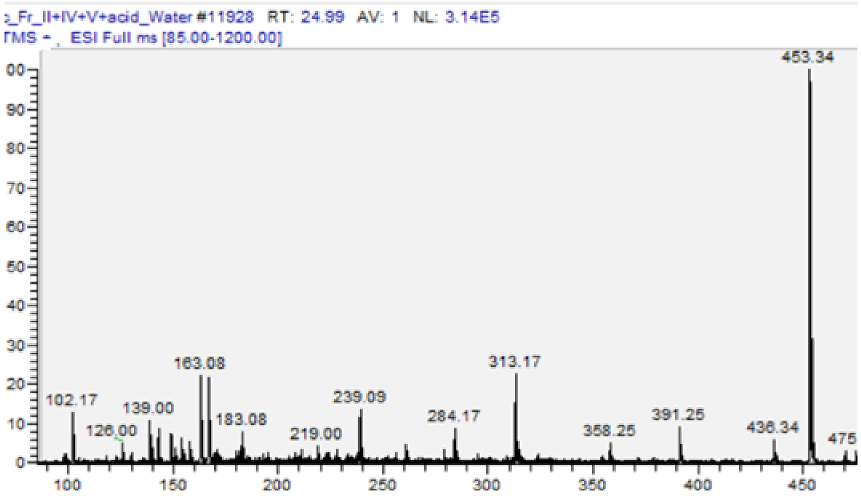

2: Pyrogallol-O-methyl-Galloyl-Glucosude

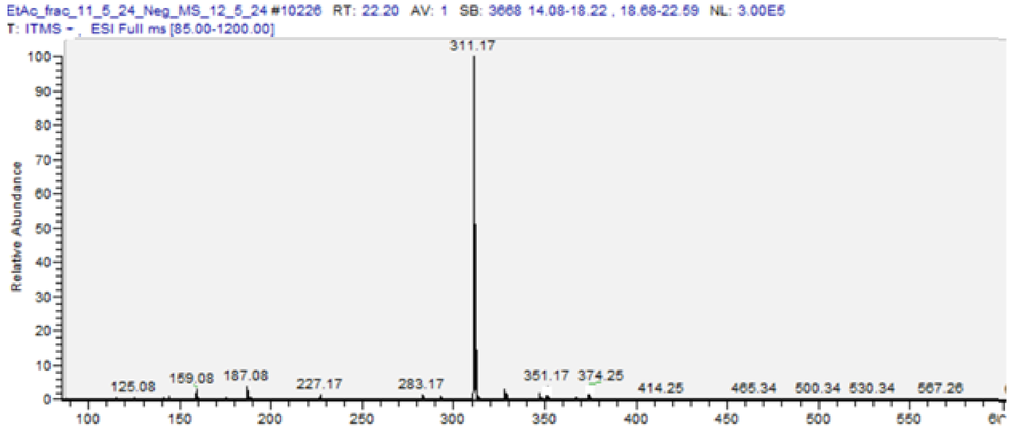

3: 7,3’-O-dimethyl-Quercetin

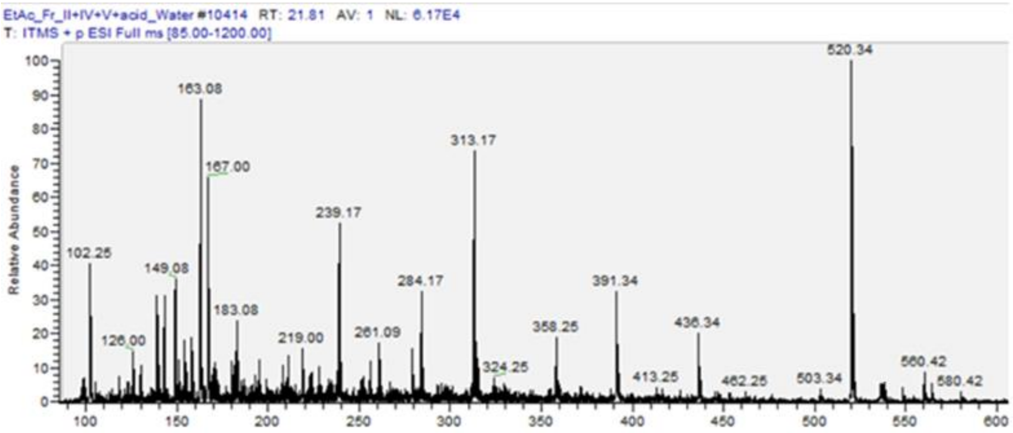

4: 7-O-Glc-3’-O- CH_3-3-O-Acetyl-Quercetin

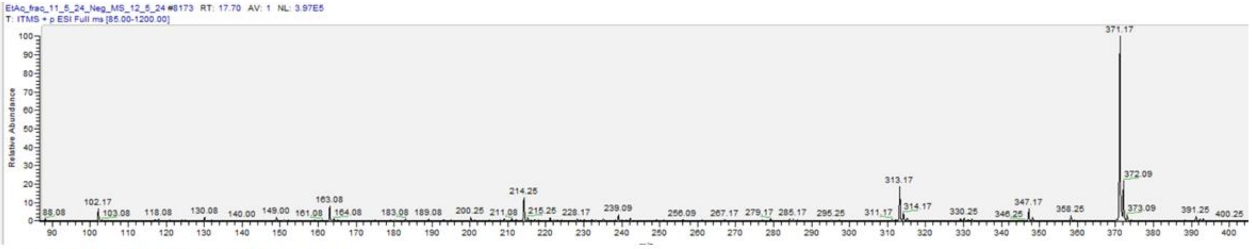

6: 7, 3’-O- CH_3-3-O-Acetyl-Quercetin

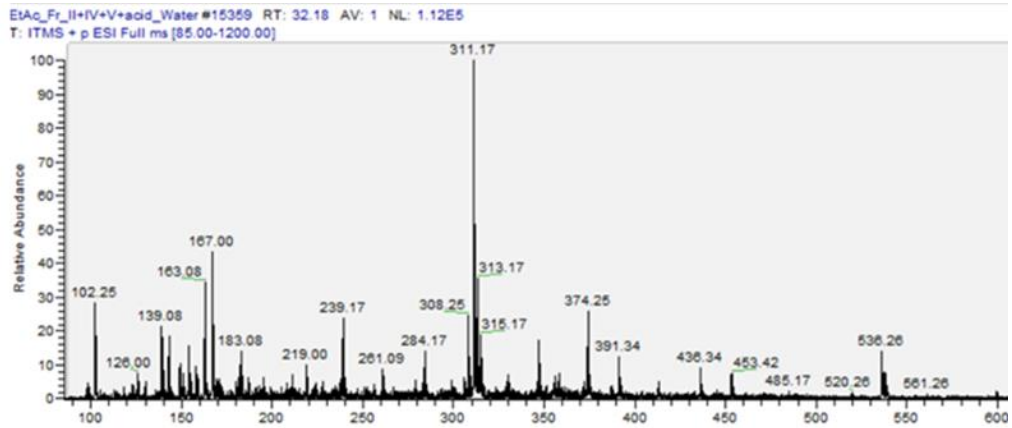

8: 7-O-Glu-3',5'-O-Dimethyl-1-0-Acetyl-Quercetin

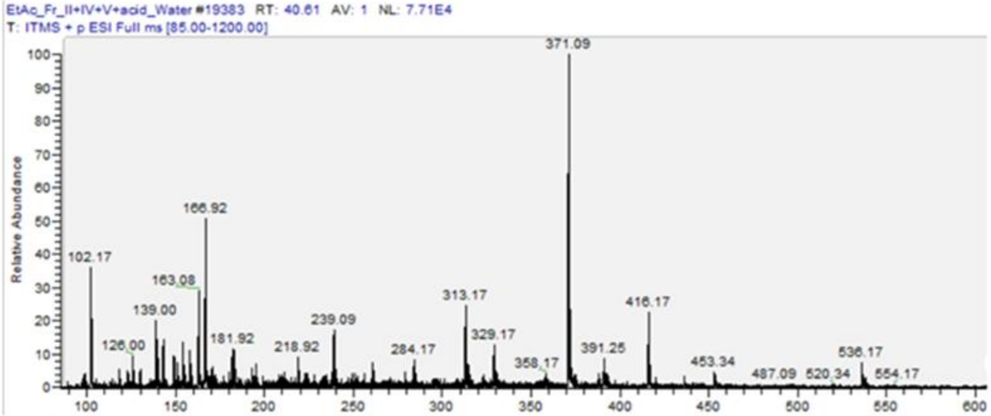

9: 7-O-Rha-3'-O-Methyl-1-0-Methyl-Quercetin

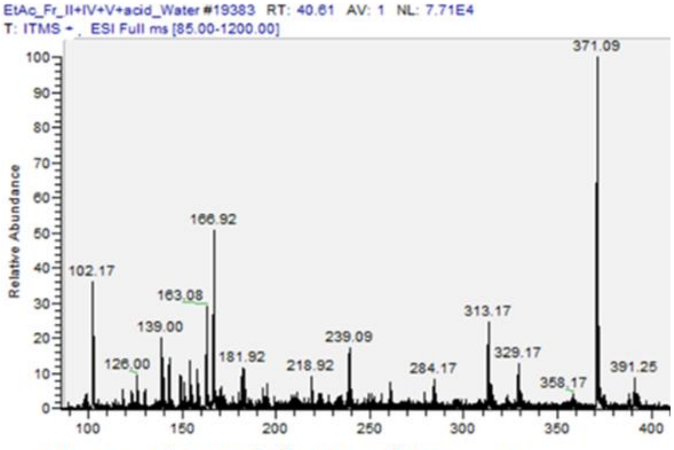

10: 7-O-3'-O-Methyl-3-Acetyl-Quercetin

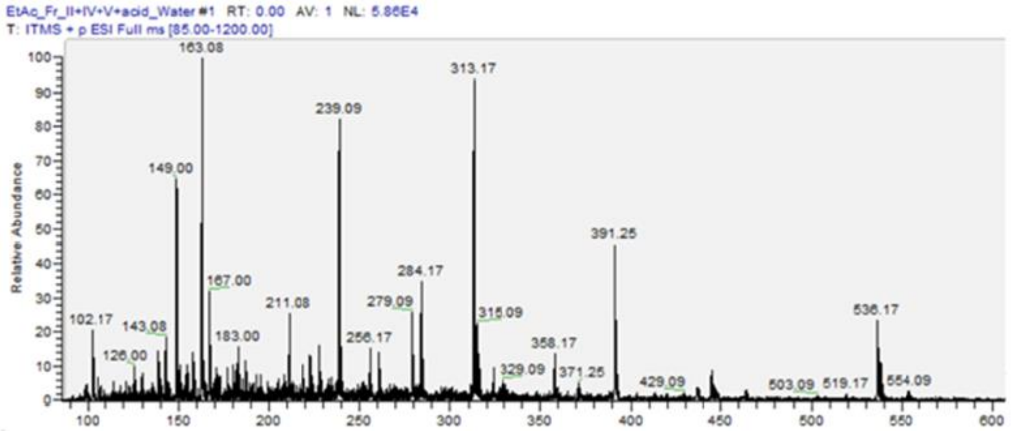

11 and 12: 7-O-Glu-3'-O-Methyl-3-acetyl-Quercetin

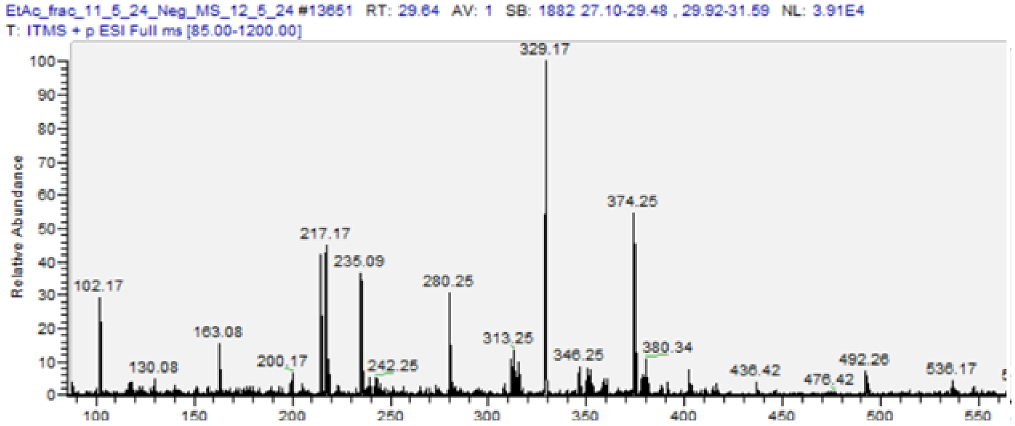

13: 7-O-Glu-3'-O-Methyl-1-Acetyl-Quercetin

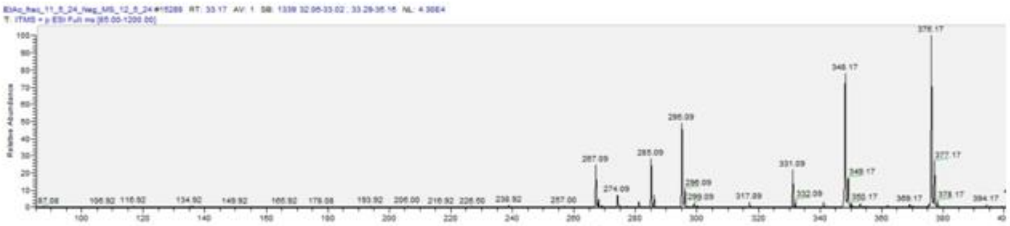

14: 7, 3'- dimethyl-3-O-Acetyl-Quercetin

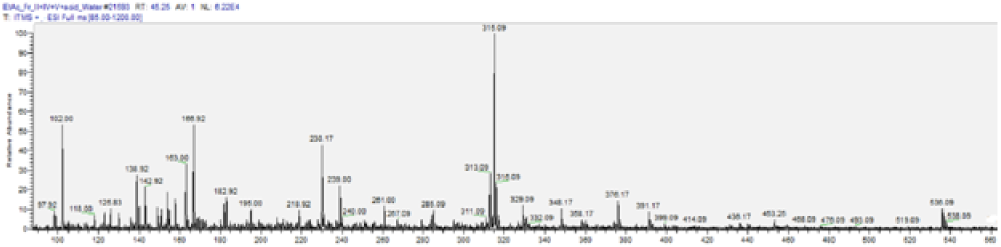

16: 7-O-Glu-3’, 5-O-Dimethyl-1-Acetyl-Flavanol

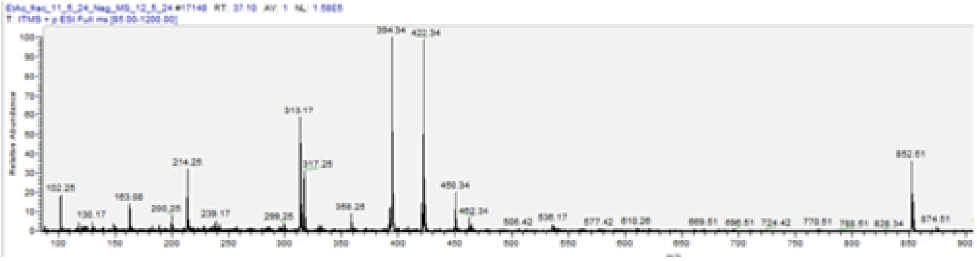

18: 7-O-Xy-Rhm–Glc-3-O-acetyl Miricetin

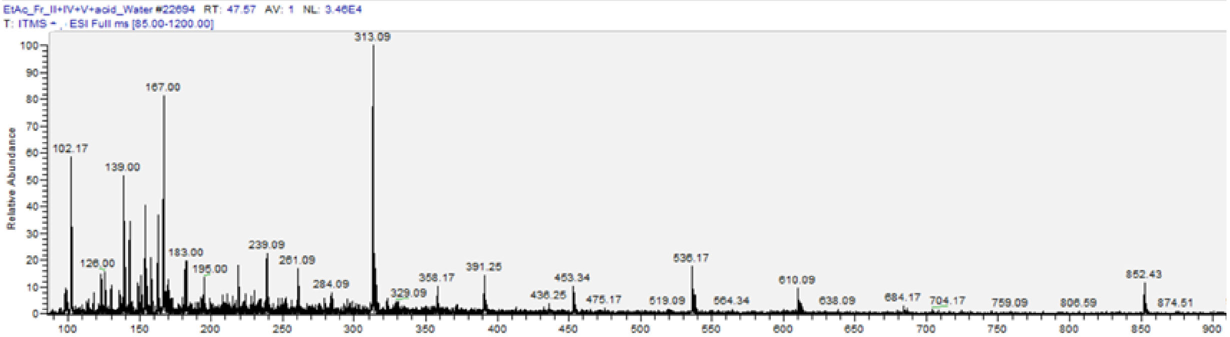

18: 3’-O-methyl-Quarcetin-3-O-Rha-7²-O-3’²,5’²-dimethylQuarcetin-3-O-ethylacetate M 853.431

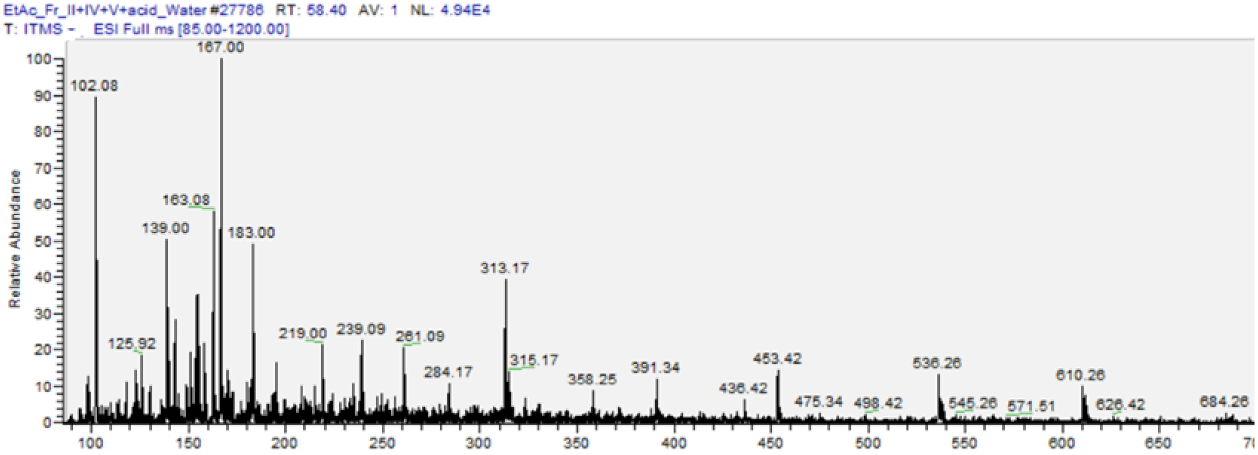

19: Rha- Rha-7-O-3’,5’-dimethylQuarcetin-3-O-ethylacetate

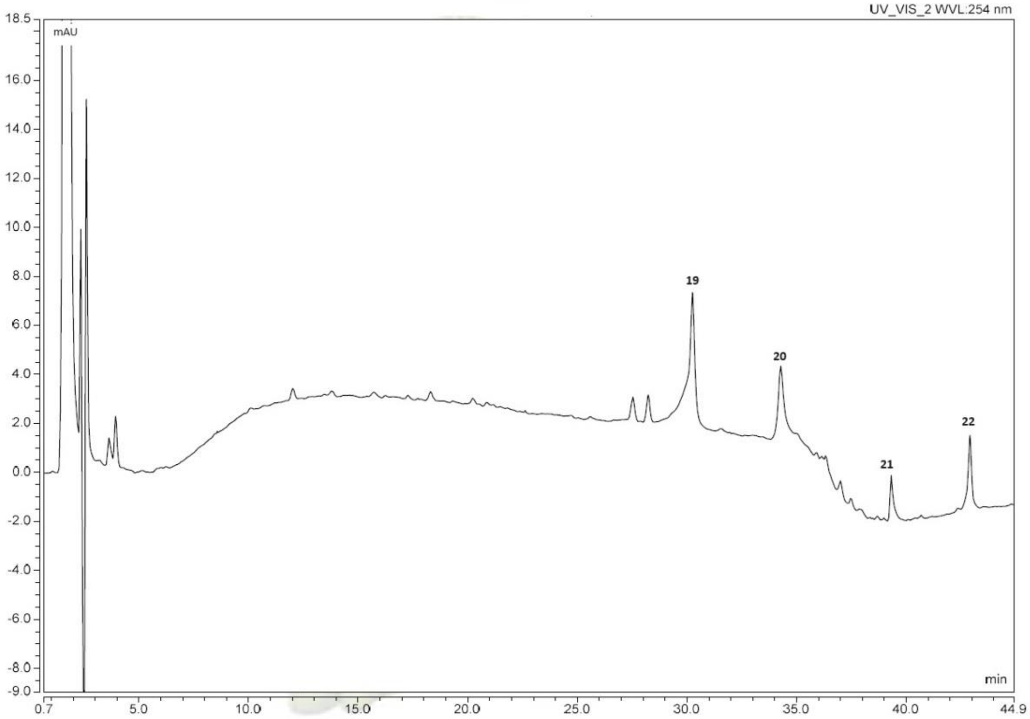

Figure S3 - Chromatogram of *C. lechleri* twigs Soxhlet water extract

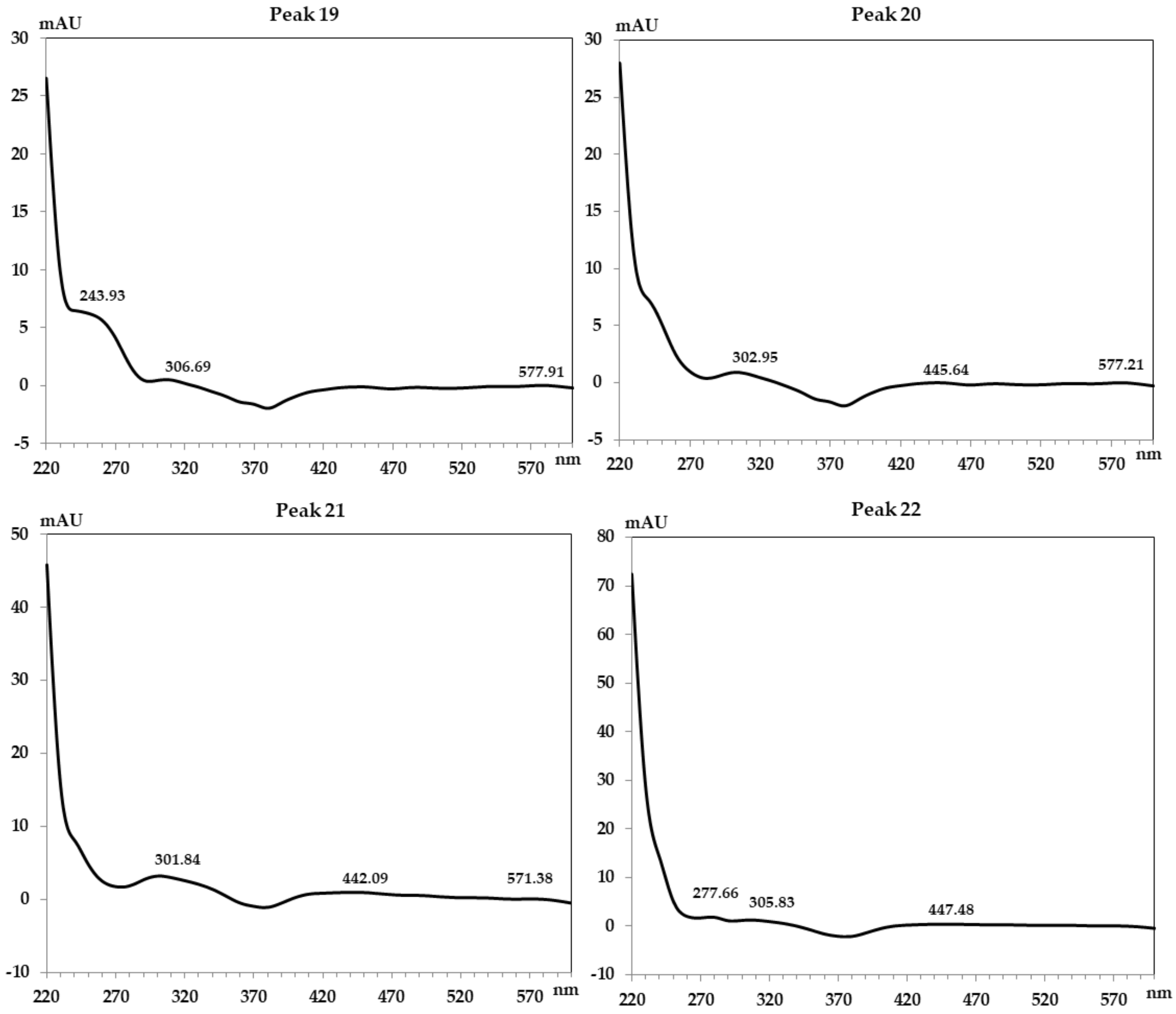

Figure S4 - UV-Vis spectra of compounds from *C. lechleri* twigs Soxhlet water extract.

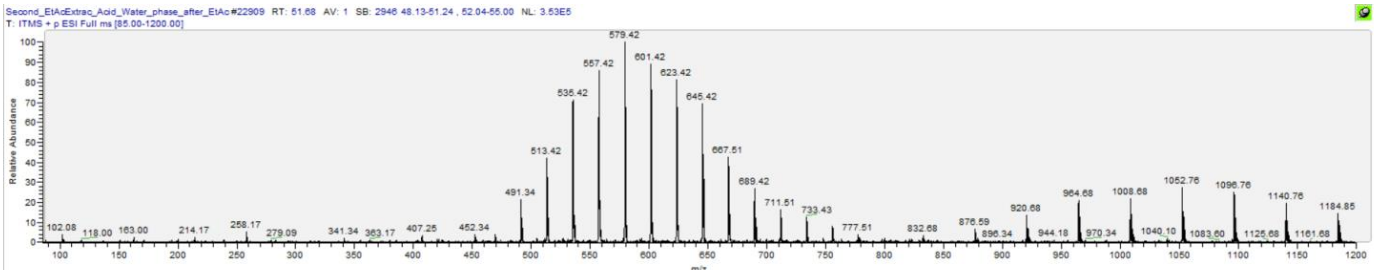

Figure S5 - MS spectrum of peak with tR 34 and 43 min - Oligomers and polymers (molar mass ranging from 500 to 3000).

Section 3.4 - Total phenolic content, antioxidant activity, and total flavonoid content (TFC)

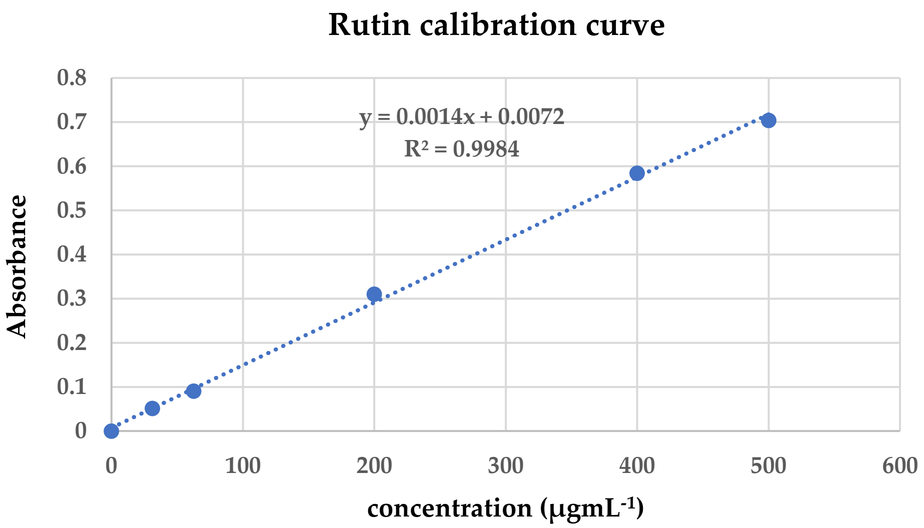

Figure S6 Calibration curve for quantification of the total flavonoid content in the *C. lechleri* extracts.
